# IFN-γ-mediated control of SARS-CoV-2 infection through nitric oxide

**DOI:** 10.3389/fimmu.2023.1284148

**Published:** 2023-12-15

**Authors:** Bruno J. de Andrade Silva, Paul A. Krogstad, Rosane M. B. Teles, Priscila R. Andrade, Jacob Rajfer, Monica G. Ferrini, Otto O. Yang, Barry R. Bloom, Robert L. Modlin

**Affiliations:** ^1^ Division of Dermatology, Department of Medicine, David Geffen School of Medicine at University of California (UCLA), Los Angeles, CA, United States; ^2^ Department of Pediatrics, David Geffen School of Medicine at UCLA, Los Angeles, CA, United States; ^3^ Department of Molecular and Medical Pharmacology, UCLA, Los Angeles, CA, United States; ^4^ Department of Urology, David Geffen School of Medicine at UCLA, Los Angeles, CA, United States; ^5^ Department of Medicine, David Geffen School of Medicine at UCLA, Los Angeles, CA, United States; ^6^ Department of Health and Life Sciences, Charles R. Drew University of Medicine and Science, Los Angeles, CA, United States; ^7^ Department of Microbiology, Immunology and Molecular Genetics, David Geffen School of Medicine at UCLA, Los Angeles, CA, United States; ^8^ Department of Immunology and Infectious Diseases, Harvard T.H. Chan School of Public Health, Boston, MA, United States

**Keywords:** interferon, T cell response, CD8 lymphocytes, nitric oxide, COVID-19, SARS-CoV-2, viral immunity

## Abstract

**Introduction:**

The COVID-19 pandemic has highlighted the need to identify mechanisms of antiviral host defense against SARS-CoV-2. One such mediator is interferon-g (IFN-γ), which, when administered to infected patients, is reported to result in viral clearance and resolution of pulmonary symptoms. IFN-γ treatment of a human lung epithelial cell line triggered an antiviral activity against SARS-CoV-2, yet the mechanism for this antiviral response was not identified.

**Methods:**

Given that IFN-γ has been shown to trigger antiviral activity via the generation of nitric oxide (NO), we investigated whether IFN-γ induction of antiviral activity against SARS-CoV-2 infection is dependent upon the generation of NO in human pulmonary epithelial cells. We treated the simian epithelial cell line Vero E6 and human pulmonary epithelial cell lines, including A549-ACE2, and Calu-3, with IFN-γ and observed the resulting induction of NO and its effects on SARS-CoV-2 replication. Pharmacological inhibition of inducible nitric oxide synthase (iNOS) was employed to assess the dependency on NO production. Additionally, the study examined the effect of interleukin-1b (IL-1β) on the IFN-g-induced NO production and its antiviral efficacy.

**Results:**

Treatment of Vero E6 cells with IFN-γ resulted in a dose-responsive induction of NO and an inhibitory effect on SARS-CoV-2 replication. This antiviral activity was blocked by pharmacologic inhibition of iNOS. IFN-γ also triggered a NO-mediated antiviral activity in SARS-CoV-2 infected human lung epithelial cell lines A549-ACE2 and Calu-3. IL-1β enhanced IFN-γ induction of NO, but it had little effect on antiviral activity.

**Discussion:**

Given that IFN-g has been shown to be produced by CD8+ T cells in the early response to SARS-CoV-2, our findings in human lung epithelial cell lines, of an IFN-γ-triggered, NO-dependent, links the adaptive immune response to an innate antiviral pathway in host defense against SARS-CoV-2. These results underscore the importance of IFN-γ and NO in the antiviral response and provide insights into potential therapeutic strategies for COVID-19.

## Introduction

The rapid emergence of severe acute respiratory syndrome coronavirus 2 (SARS-CoV-2) in late 2019 and early 2020 led to a global pandemic of coronavirus disease 2019 (COVID-19) (1). More than two years later, several vaccines are authorized or approved for large-scale immunizations in the United States to prevent COVID-19, and effective therapeutics against SARS-CoV-2 have become available (2–4). At the same time, the emerging SARS-CoV-2 variants of concern display enhanced infectivity, transmissibility, and resistance to vaccine-induced neutralization antibodies, such that widespread infections persist albeit with decreased mortality (5, 6). To overcome the challenges related to viral escape from humoral responses, there is an urgent need to understand additional host defense mechanisms against SARS-CoV-2 (7).

For over 50 years, interferons (IFNs) have been known to have antiviral activity (8). The importance of the IFNs is indicated by findings that SARS-CoV-2 suppresses the production of type I IFNs (IFN-α and IFN-β), which is associated with severe clinical outcomes (9–12). Increased levels of IFN-γ and enhanced IFN-γ gene expression were observed in convalescent COVID-19 patients, indicating the potential role for IFN-γ in the control of SARS-CoV-2 infection (13, 14). In immunocompromised individuals, treatment with IFN-γ led to the clearance of SARS-CoV-2 infection and clinical recovery of respiratory status (15–17). However, a prolonged IFN-γ response is associated with severe tissue inflammation and a poor outcome in COVID-19 patients (10, 12, 17).

Although IFN-γ exerted an antiviral activity against SARS-CoV-2 infection in the lung epithelial cell line Calu-3 (18), yet no mechanism has been identified for how this cytokine triggers an antiviral response. In general, IFN-γ is known to combat viruses via the production of nitric oxide (NO) by NO synthase 2 (*NOS2*; also known as inducible NO synthase, iNOS) (19, 20). Of the IFNs, IFN-γ is the most effective inducer of *NOS2* gene expression (21, 22). To gain insight into immune pathways that might contribute to host defense against COVID-19 infection, we investigated whether IFN-γ induction of an antiviral activity against SARS-CoV-2 in cell line culture is linked to the production of NO.

## Materials and methods

### Cell lines and virus strains

Vero E6 (CRL-1586) and Calu-3 cells (HTB-55) were purchased from ATCC. A549 cell line (BEI Resources #NR-53821) stably expressing ACE2 (A549-ACE2) (23) was a kind gift of Dr. Bryan Bryson (Ragon Institute of MGH, MIT, and Harvard). Cells were cultured at 37°C with 5% CO_2_ humidified conditions in DMEM containing 10% (or 20% for Calu-3 cells) fetal bovine serum (FBS, Seradigm), 2 mM L-glutamine, 100 U/mL penicillin and 100 mg/mL streptomycin, and 1 mM sodium pyruvate (complete medium). The SARS-CoV-2 clinical isolate USA-WA1/2020 (24) was obtained through BEI Resources (NR-52281) and amplified in Vero E6 cells. The USA-WA1/2020 clone expressing the reporter protein mNeonGreen (SARS-CoV-2-mNG) was obtained from the University of Texas Medical Branch at Galveston through a material transfer agreement (25, 26). The fluorescent SARS-CoV-2-mNG clone can be provided by the World Reference Center for Emerging Viruses and Arboviruses (WRCEVA) pending scientific review and a completed material transfer agreement. Requests for the SARS-CoV-2-mNG strain should be submitted to: P.-Y. Shi and S. Mattamana/WRCEVA. Viral titers were determined in Vero E6 cells by established TCID_50_ assay (24, 27). The key reagents and resources used in this study are listed in the **Supplementary Table 1**. All experiments involving SARS-CoV-2 isolate USA-WA1/2020 were carried out in the UCLA BSL3 High-Containment Facility, between April 2020 and April 2021, before the emergence of SARS-CoV-2 variants Delta and Omicron. Although the Delta variant was first reported in the United States in March 2021, it only became the dominant strain by July 2021. The Delta variant (lineage B.1.617.2) was first isolated in the United States by the end of April 2021 (isolates MD-HP05285/2021 and MD-HP05647/20210) and only became available as a resource in August 2021 (BEI Resources). As for the Omicron variant (lineage B.1.1.529), the first reported case in the United States date of December 1, 2023, with the variant becoming available for research later that month (MD-HP20874/2021 and HI-CDC-4359259-001/2021, BEI Resources).

### Cytokine treatment and viral infection

Cells were plated overnight in complete medium containing 10% FBS. After that, cells were washed twice with complete medium containing phenol red-free DMEM (Gibco) instead of the regular DMEM and incubated in the same medium in the presence of IFN-α 2a (PBL Assay Science) at 10, 50, 100, 200, 400, or 1000 U/mL; IFN-β 1b (PBL Assay Science) at 10, 50, 100, 200, 400, or 1000 U/mL; IFN-λ1 (Peprotech) at 0.1, 0.5, 1, 2, 4, or 10 ng/mL; IFN-γ (BD Pharmigen) at 10, 50, 100, 200, 400, or 1000 U/mL; IL-1β (Gibco) at 1.25, 2.5, 5, or 10 ng/mL; or cytokine combinations of IFN-γ and IL-1β at 50/1.25, 100/2.5, 200/5, or 400/10, respectively. We used previously published concentrations as a starting point reference for cytokine titrations (18, 20, 28). Forty-eight hours posttreatment, cells washed twice with reduced-serum medium Opti-MEM (Gibco) and infected with the SARS-CoV-2 strains described above (MOI of 0.1 and 1 for Vero E6; or MOI of 1 for A549-ACE2 and Calu-3 cells) for 1 h at 37°C using 0.2 mL of serum-free media as final volume. To account for variations in cell numbers after a 48-hour period, we adjusted our experimental conditions based on each cell line’s specific doubling time: 24 h for Vero E6 cells, 22 h for A549-ACE2 cells, and 48 h for Calu-3 cells. We determined the final seeding density by counting the cells after 48 h of treatment to prevent excessive cell density. For mock infection, 0.2 mL of Opti-MEM was added per well. The viral inoculum was spread by gently tilting the plate sideways every 15 minutes. Lastly, the inoculum was removed, and cells were washed twice and cultured in phenol red-free complete medium for an additional 24 h.

### NOS inhibitors

Cytokine treatment was carried out in the presence or absence of the pharmacologic inhibitors of iNOS N6-(1-iminoethyl)-L-lysine (L-NIL; 1 mM), L-N^G^-Nitroarginine-methyl ester (L-NAME; 2 mM), or the inactive enantiomer D-N^G^-Nitroarginine-methyl ester (D-NAME; 2 mM). We chose the concentration of iNOS inhibitors based on published papers using activated cells (29–32). All chemicals were purchased from Cayman Chemical.

### Viral titer by median tissue culture infectious dose (TCID_50_) assay

Vero E6 cells were seeded overnight in 96-well plates at a density of 7x10^3^ cells per well. Next, culture media samples harvested at 24 h post-infection were subjected to 10-fold serial dilutions (10^1^ to 10^7^) in Opti-MEM and inoculated onto Vero E6 cells. One-hour post-infection, medium was replaced by DMEM supplemented with 2% FBS and cells were incubated for 3 days at 37°C with 5% CO_2_. Subsequently, each inoculated well was evaluated for the presence or absence of viral CPE (33) and the percent of infected dilutions immediately above and below 50% were determined. TCID_50_ (24, 27) was calculated based on the Spearman-Karber method (34).

### Virus detection by live-cell imaging

Vero E6 or A549-ACE2 cells were seeded overnight in 24-well plates (Corning) at a density of 2x10^4^ cells per well. After cytokine treatment and infection with SARS-CoV-2-mNG (MOI of 0.1 for Vero E6; or MOI of 1 for A549-ACE2), cells were washed twice and imaged in FluoroBrite DMEM media (Gibco) by live-cell fluorescence microscopy using the FITC filter set on a Leica DM IRB inverted modulation contrast microscope. Image acquisition was carried out with a FLUOTAR 10x objective controlled by Leica Microsystems Application Suite X software. For A549-ACE2 experiments, NucBlue nuclear staining for live cells (Invitrogen) was added (2 drops per mL of media) in the last 15 minutes of incubation and detected through a DAPI filter set. In some experiments, Vero E6 cells were infected with SARS-CoV-2 at a MOI of 1 to visualize the amount of virus required to produce CPE in 50% of inoculated tissue culture cells (TCID_50_ assay). Representative images of cytokine treated cells displaying viral CPE (in 24-well plates) were taken at 96 h post-infection (to match the time point where the 96-well plates containing cell supernatants were scored) with a HI PLAN PH1 10x objective on a Leica DMi1 inverted phase contrast digital microscope. Leica Microsystems Application Suite software was used for image acquisition.

### Virus detection by flow cytometry

Vero E6 were seeded overnight in 24-well plates at a density of 2x10^4^ cells per well. Following cytokine treatment and viral infection with SARS-CoV-2-mNG (MOI = 0.1), cells were washed, dissociated with 0.25% Trypsin-EDTA (Gibco), and then fixed for 30 min with 4% PFA (Thermo Scientific) at room temperature. Live Vero E6 cells were acquired according to FSC-SSC parameters and doublet exclusion on a SORP LSRII Analytic Flow Cytometer using the FACSDiva software version 8.0.2 (BD Biosciences). FlowJo software version 10.7.1 (BD Biosciences) was used to analyze flow cytometry data, which were used to generate the dose-response curves.

### NO detection by DAF-FM staining and live-cell imaging

The NO indicator DAF-FM diacetate (Invitrogen) was used to quantify NO production (35, 36). Vero E6 cells were plated overnight in Millicell EZ 8-well glass slides (Millipore) at a density of 1x10^4^ cells per well. After cytokine treatment and infection with SARS-CoV-2 (MOI = 0.1), cells were washed twice and incubated in warm phenol red-free DMEM (without serum) supplemented with 5 µM DAF-FM diacetate for 30 min at 37°C. Next, Vero E6 cells were washed 3 times with warm phenol red-free DMEM and incubated for an additional 15 min to allow complete de-esterification of the intracellular diacetates. Finally, cells were washed twice and immediately imaged in FluoroBrite DMEM media as described above for SARS-CoV-2-mNG. Image acquisition was carried out with a FLUOTAR 20x objective controlled by Leica Microsystems Application Suite X software.

### NO detection by DAF-FM staining and flow cytometry

Vero E6 were seeded overnight in 24-well plates at a density of 2x10^4^ cells per well. Following cytokine treatment and viral infection with SARS-CoV-2 (MOI = 0.1), cells were washed and stained with 1 µM DAF-FM diacetate as described above. Next, cells were dissociated with 0.25% Trypsin-EDTA and fixed for 30 min with 4% PFA at room temperature. Vero E6 cells were then washed once with 1X PBS and twice with warm phenol red-free DMEM, and immediately acquired on a SORP LSRII Analytic Flow Cytometer by gating on live, single cells, according to FSC-SSC parameters. FACSDiva software version 8.0.2 was used for acquisition. FlowJo software version 10.7.1 was used to analyze flow cytometry data, which were used to generate the dose-response curves. For uninfected Vero E6 or A549-ACE2 cells, acquisition of live, single cells, was performed immediately after DAF-FM diacetate staining without fixation on either a SORP LSRII Analytic Flow Cytometer or a SORP LSRFortessa X-20 (BD Biosciences). In some experiments, Vero E6 cells were additionally treated with IFN-γ at 800 or 1600 U/mL; IL-1β at 20 or 40 ng/mL; or cytokine combinations of IFN-γ and IL-1β at 800/20 or 1600/40, respectively, and double stained with DAF-FM diacetate and SYTOX Red dead-cell indicator (Invitrogen).

### Antibodies

Monoclonal antibodies and their corresponding isotype controls used were the following: Alexa Fluor 594 anti-NOS2/iNOS (1 µg/test; BioLegend #696804), Alexa Fluor 594 rat IgG2bκ isotype (1 µg/test; BioLegend #400661), rat anti-mouse/human iNOS CXNFT (10 µg/mL; eBioscience #14-5920-82) (37), purified rat IgG2aκ isotype (10 µg/mL; BD Biosciences #553927), anti-SARS-CoV-2 Nucleocapsid (5.6 µg/mL; Sino Biological #40143-MM05), and IgG1κ isotype from murine myeloma (5.6 µg/mL; Sigma-Aldrich #M9269). Conjugated secondary antibodies used (1:1000) were the following: goat anti-mouse IgG1 Alexa Fluor 488 (Invitrogen #A-21121), goat anti-mouse IgG1 Alexa Fluor 568 (Invitrogen #A-21124), goat anti-rat IgG Alexa Fluor 568 (Invitrogen #A-11077), and goat anti-rat IgG Alexa Fluor 647 (Invitrogen #A-21247).

### Confocal microscopy

Vero E6 (2x10^4^ cells/well), A549-ACE2 (2x10^4^ cells/well), or Calu-3 cells (4x10^4^ cells/well) were seeded overnight in Millicell EZ 4-well glass slides (Millipore). Following cytokine treatment and viral infection, cells were washed twice with 1X PBS and fixed for 30 min with 4% PFA at room temperature. In some experiments, cells were stained with DAF-FM diacetate (as described above) before fixation. Next, cells were washed again, blocked with 5% normal goat serum (Vector Laboratories) in 1X PBS containing 0.05% saponin (Sigma-Aldrich) for 20 minutes, and then immunolabeled with indicated primary antibodies for 1 h at room temperature. Following washing, cells were stained with secondary antibodies for 1 h in the dark, washed, and mounted with ProLong Gold Antifade with, or without DAPI (Invitrogen), where NucBlue dye was used instead. Cells were examined using a Leica TCS SP8 Digital LightSheet Laser Scanning Confocal Microscope at the Advanced Light Microscopy and Spectroscopy Laboratory, California NanoSystems Institute at UCLA. Image acquisition was carried out with the CS2 63x or 100x/1.4 oil objectives controlled by Leica Microsystems Application Suite X software.

### Intracellular flow cytometry staining

Vero E6 cells were seeded overnight in 24-well plates at a density of 2x10^4^ cells per well. Following cytokine treatment and viral infection with SARS-CoV-2-mNG (MOI = 0.1), cells were washed and incubated with normal human serum (GeminiBio) for 10 min. Next, cells were washed again, dissociated with 0.25% Trypsin-EDTA and fixed for 30 min with 4% PFA at room temperature. Cells were then suspended in permeabilization buffer (1X PBS containing 0.5% saponin and 10% FBS) for 15 min and stained with fluorescently labeled iNOS-AF594 antibody or matching isotype for 1 h at room temperature in the dark. Following two washes with FACS buffer (1X PBS with 2% FBS), Vero E6 cells were resuspended in FACS buffer containing 2% PFA and acquired on a SORP LSRII Analytic Flow Cytometer by gating on live, single cells, according to FSC-SSC parameters. FACSDiva software version 8.0.2 was used for acquisition. FlowJo software version 10.7.1 was used to analyze flow cytometry data, which were used to generate the correlation graph.

### Image quantification and scientific illustrations

The image calculator tool by ImageJ (Fiji) software (38) was used to measure fluorescence intensity (mean gray value) as previously described (39, 40). Colocalization and Analyze Particles built-in functions of ImageJ were used for colocalization analysis as described before (39, 40). Colocalization of SARS-CoV-2 with cell markers was carried out with infected cells only. For both quantifications, a minimum of 100 cells per sample were scored for each experiment, unless stated otherwise. Schematic illustrations were created with BioRender.com.

### Statistical analysis

Statistical analysis and graphing were undertaken with GraphPad Prism software version 9.1.0 (Dotmatics). Statistics reported are of entire series of experiments and described as mean ± the standard error (SEM). For comparison between three or more groups with matched or repeated data, we used repeated measures one-way or two-way ANOVA with the Geisser–Greenhouse correction, in addition to Tukey’s multiple comparisons test with individual variances computed for each comparison. For data without matching or pairing, we used a mixed-effects model (REML) with the Geisser–Greenhouse correction and Tukey’s multiple comparisons test. A nonlinear regression model (inhibitor vs. normalized response – variable slope) was used to calculate the IC_50_ values and Hillslopes. For comparisons involving two groups, an unpaired Mann-Whitney test was performed. Pearson correlation coefficient was used to measure the linear correlation between two sets of data. A *P* value < 0.05 was considered statistically significant.

## Results

### IFN-γ-mediated antiviral activity against SARS-CoV-2 in Vero E6 cells

**Figure 1:**
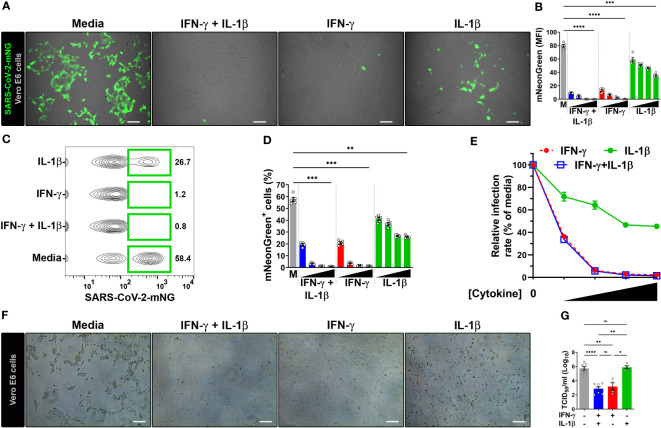
IFN-γ treatment reduces SARS-CoV-2 infection in Vero E6 cells. **(A, B)** Representative live-cell imaging analysis of Vero E6 cells pretreated with increasing concentrations of IFN-γ (50, 100, 200, 400 U/mL), IL-1β (1.25, 2.5, 5, or 10 ng/mL), or IFN-γ in combination with IL-1β (50/1.25, 100/2.5, 200/5, or 400/10, respectively) for 48 h, and infected with SARS-CoV-2-mNG (MOI = 0.1). Displayed images represent cytokines at the highest concentrations **(A)**. Scale bars, 100 µm. **(B)** The MFI of SARS-CoV-2-mNG (green) in Vero E6 cells was quantified with ImageJ software. M, media. Data are means ± SEM (*n* = 5). Data were analyzed by one-way ANOVA followed by Tukey’s *post hoc* test (***P < 0.001 and ****P < 0.0001). All cytokine concentrations were significantly different from the media control. **(C–E)** Flow cytometry data shown are concatenated FCS files **(C)** representing infected Vero E6 cells treated as described in **(A)**. **(D)** FlowJo software was used to determine the % of Vero E6 cells infected with SARS-CoV-2-mNG. M, media. Data are means ± SEM (*n* = 5). Data were analyzed by one-way ANOVA followed by Tukey’s *post hoc* test (**P < 0.01 and ***P < 0.001). All cytokine concentrations were significantly different from the media control. **(E)** Dose-response curves of mNG signal inhibited by cytokines treatment. **(F, G)** Vero E6 cells were treated with IFN-γ (400 U/ml), IL-1β (10 ng/mL), or IFN-γ in combination with IL-1β (400/10, respectively) for 48 h, and infected with SARS-CoV-2 (MOI = 1). Images depict the CPE development between untreated (media) and treated cells **(F)**. Scale bars, 100 µm. Progeny virus titers were determined by TCID_50_ assay **(G)**. Data are means ± SEM of at least four independent experiments. Data were analyzed by mixed-effects model with the Geisser–Greenhouse correction and Tukey’s *post hoc* test (*P < 0.05, **P < 0.01, and ****P < 0.0001; ns, not statistically significant).

Due to the well-established antiviral effect on the replication cycle of SARS-CoV-1 (20), achieved through the 48 h pretreatment of Vero E6 cells with IFN-γ (400 U/mL) together with IL-1β (10 ng/mL), we aimed to investigate whether these cytokines could elicit a similar antiviral response in SARS-CoV-2-infected cells. Vero E6 cells, an epithelial cell line isolated from the kidney of a normal African green monkey, are widely used as a model to study epithelial cell infection by SARS-CoV-2 and the associated host defense response due to their high expression of ACE2 receptor and inability to produce type I IFN (1, 23, 24, 41), a known inducer of antiviral responses, although they can respond to exogenous treatment with human IFNs (11, 20, 25, 28, 42). To do so, we subjected Vero E6 cells to a 48-hour co-treatment with IFN-γ and IL-1β before exposing them to SARS-CoV-2 infection. For our experiments, we employed an infectious clone derived from the USA-WA1/2020 isolate (25, 26) expressing a mNeonGreen fluorescent protein (SARS-CoV-2-mNG) at a MOI of 0.1, and subsequently we performed live-cell imaging at 24 h post-infection (**Supplementary Figure 1A**). We observed a dose-dependent reduction of mNG fluorescence signal when infected Vero E6 cells were pretreated with IFN-γ and IL-1β (**Figures 1A, B**; **Supplementary Figure 1B**). Given that IFN-γ and IL-1β can mount antiviral responses by themselves (21, 43), we sought to determine whether these cytokines could individually control SARS-CoV-2 infection in Vero E6 cells. Fluorescence microscopy showed a dose-response antiviral effect by both IFN-γ and IL-1β alone (**Figures 1A, B**; **Supplementary Figure 1B**), although the virus level was more prominently reduced in cells treated with IFN-γ, to similar levels observed in Vero E6 cells stimulated with IFN-γ and IL-1β simultaneously (**Figures 1A, B**; **Supplementary Figure 1B**).

We next performed flow cytometry to monitor SARS-CoV-2 infection. Similar to the live-cell imaging experiments, IFN-γ treatment in combination with IL-1β was able to reduce not only the number of infected cells (**Figures 1C, D**; **Supplementary Figure 1C**) but also the amount of intracellular viral replication (**Supplementary Figure 1D**) in a dose-dependent manner. The virus level was also reduced in Vero E6 cells singly treated with IFN-γ and IL-1β, with IFN-γ treatment showing similar results to the combination of IFN-γ and IL-1β (**Figures 1C, D**; **Supplementary Figure 1C, D**). We used a nonlinear regression model to determine the half maximal inhibitory concentration (IC_50_) of SARS-CoV-2 replication. Analysis of flow cytometry experiments revealed an IC_50_ of 41.3 U/ml and 5.8 ng/ml for IFN-γ and IL-1β, respectively. Steeper dose-response inhibitory curves were observed for IFN-γ (Hillslope = -2.96) and IFN-γ together with IL-1β (Hillslope = -2.94), which overlapped with one another at all segments. However, IL-1β treatment alone (Hillslope = -0.58) exhibited a shallow curve (**Figure 1E**; **Supplementary Table 2**).

To further confirm the antiviral activity of IFN-γ and IL-1β on wild-type virus, we evaluated the inhibition of the virus infectious cycle using the median tissue culture infective dose (TCID_50_) assay. We pretreated Vero E6 cells with IFN-γ, IL-1β, or IFN-γ in combination with IL-1β, and then infected with a ten-fold larger inoculum of SARS-CoV-2 USA-WA1/2020 strain (MOI = 1) than our previous experiments (MOI = 0.1). This strain was isolated from the first COVID-19 patient diagnosed in the US (24). We evaluated cultures for a viral-induced cytopathic effect as evident by swelling and clumping of cells (24, 27, 33). A striking cytopathic effect was observed in Vero E6 cells incubated with media, indicating that the viral replication and associated cell damage persisted until 96 h post-infection, while the cells cultured with IFN-γ or IFN-γ plus IL-1β cleared the virus and showed little cytopathology at 96 h post-infection (**Figure 1F**). However, a severe cytopathic effect was observed in Vero E6 cells treated with IL-1β only (**Figure 1F**). Quantification of viral release to supernatants harvested at 24 h post-infection revealed ~350-fold lower virus titers in cells pretreated with IFN-γ and ~700-fold lower virus titers in cells pretreated with IFN-γ plus IL-1β in comparison to media alone (**Figure 1G**). Treatment with IL-1β alone had no effect on the viral load (**Figure 1G**). Collectively, the results indicate strong antiviral activities for IFN-γ or IFN-γ in combination with IL-1β against SARS-CoV-2 in Vero E6 cells.

### IFN-γ-mediated activation of nitric oxide pathway in SARS-CoV-2-infected cells

We next investigated whether the antiviral activity induced by IFN-γ was associated with NO production. We used a cell-permeant, NO-reactive green fluorescent dye, DAF-FM diacetate, to measure NO production in Vero E6 cells by flow cytometry and microscopy (35, 36). Pretreatment with IFN-γ alone resulted in the elevation of NO levels in Vero E6 cells, but stimulation with IL-1β had no effect (**Supplementary Figure 2A, B**). Treatment of uninfected Vero E6 cells with IFN-γ and IL-1β showed a dose-dependent production of NO at 48 h (**Supplementary Figure 2A, E**). These findings indicate a synergistic effect when IL-1β was added in conjunction with IFN-γ as compared to the sum of NO production when the two cytokines were added individually (**Supplementary Figure 2C**). In addition, we used SYTOX Red dead cell staining by flow cytometry to determine whether the effect of the cytokines or the NO production induced by them were associated with cytotoxicity (44, 45). Neither IFN-γ, IL-1β, or IFN-γ in combination with IL-1β, induced cell death in Vero E6 cells, whereas IFN-γ or IFN-γ in combination with IL-1β, but not IL-1β, induced NO production in the same cultures (**Supplementary Figure 2F, G**). Treating with increased concentrations of the cytokines did not induce cytotoxicity, whereas treatment with IFN-γ, or IFN-γ in combination with IL-1β, but not IL-1β, induced a dose-dependent increase of NO production (**Supplementary Figure 2F, G**).

**Figure 2:**
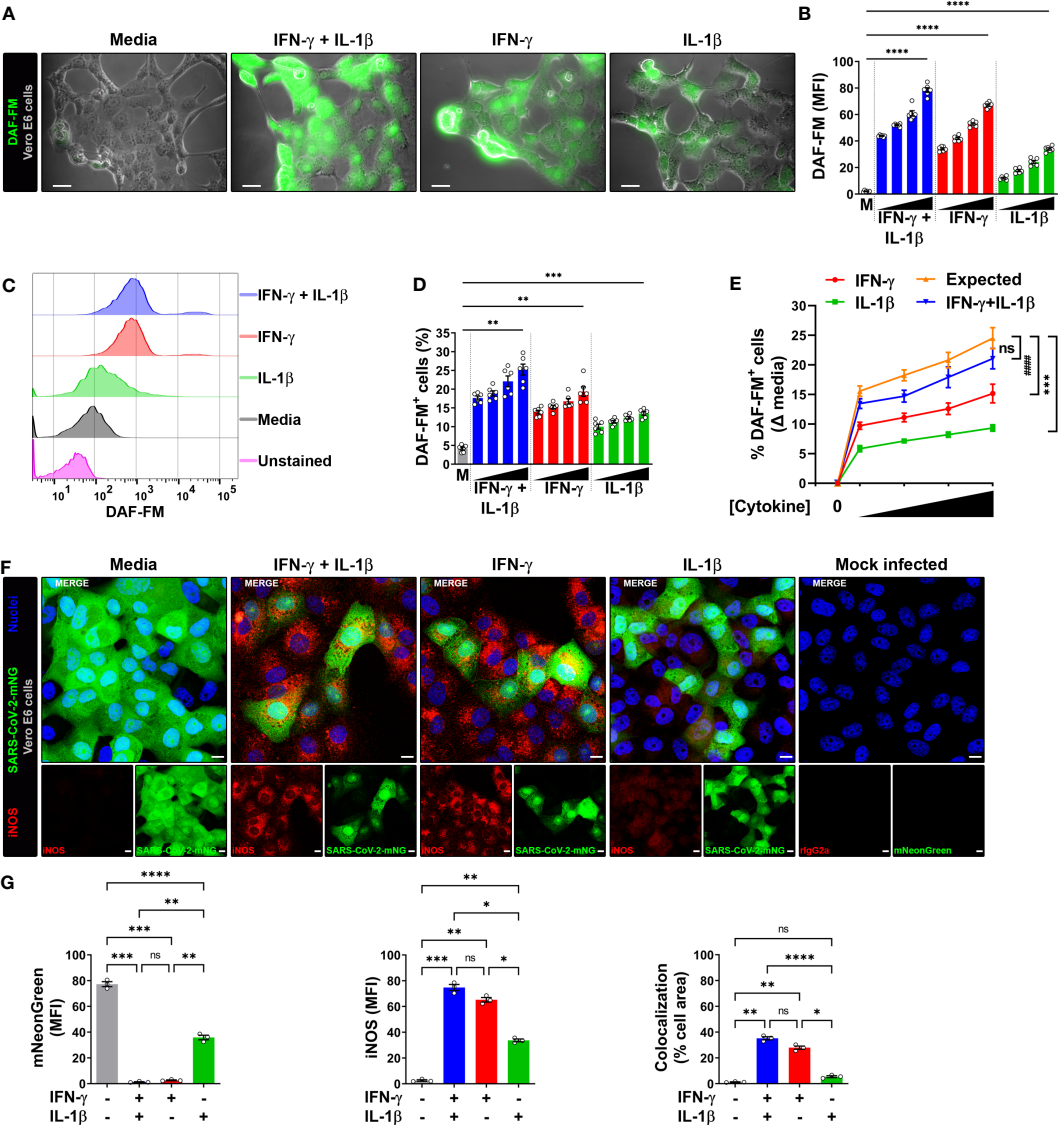
IFN-γ induces nitric oxide in SARS-CoV-2-infected Vero E6 cells. **(A, B)** Representative live-cell imaging analysis of Vero E6 cells pretreated with increasing concentrations of IFN-γ (50, 100, 200, 400 U/mL), IL-1β (1.25, 2.5, 5, or 10 ng/mL), or IFN-γ in combination with IL-1β (50/1.25, 100/2.5, 200/5, or 400/10, respectively) for 48 h, infected with SARS-CoV-2 (MOI = 0.1), and labeled with nitric oxide indicator DAF-FM (green). Displayed images represent cytokines at the highest concentrations **(A)**. Scale bars, 50 µm. **(B)** The MFI of DAF-FM in Vero E6 cells was quantified with ImageJ software. M, media. Data are means ± SEM (*n* = 6). Data were analyzed by one-way ANOVA followed by Tukey’s *post hoc* test (*P < 0.05 and **P < 0.01). All cytokine concentrations were significantly different from the media control. **(C–E)** Flow cytometry analysis of DAF-FM. Infected Vero E6 cells were treated and stained as described in **(A)**. **(D)** FlowJo software was used to determine the % of infected Vero E6 cells positive for DAF-FM. M, media. Data are means ± SEM (*n* = 6). Data were analyzed by one-way ANOVA followed by Tukey’s *post hoc* test (**P < 0.01 and ***P < 0.001). All cytokine concentrations were significantly different from the media control. **(E)** Dose-response curves of DAF-FM signal induced by cytokines treatment. Data are means ± SEM (*n* = 6). Data were analyzed by two-way ANOVA followed by Tukey’s *post hoc* test (***P < 0.001 and ^####^ *P* < 0.0001; ns, not statistically significant). **(F, G)** Vero E6 cells were treated with IFN-γ (400 U/ml), IL-1β (10 ng/mL), or IFN-γ in combination with IL-1β (400/10, respectively) for 48 h, mock infected or infected with SARS-CoV-2-mNG (green; MOI = 0.1), and then stained with anti-iNOS (red) Ab or isotype control and observed by fluorescent confocal microscopy **(F)**. Yellow denotes colocalization between green and red channels. Scale bars, 10 µm. The MFI of SARS-CoV-2-mNG (left), iNOS (middle), and the two-color colocalization (right) in infected Vero E6 cells was quantified with ImageJ software **(G)**. Data are means ± SEM (*n* = 6). Data were analyzed by one-way ANOVA followed by Tukey’s *post hoc* test (***P < 0.001 and ****P < 0.0001).

Infection of Vero E6 cells with SARS-CoV-2 showed a morphologic cytopathic effect by microscopy as has been reported (24, 27, 33) (**Figure 2A**; **Supplementary Figure 3A, B**). As measured by live microscopy, treatment of infected cells with IFN-γ or IL-1β alone induced a dose-dependent increase in NO production, with greater production observed when IFN-γ was added in combination with IL-1β (**Figures 2A, B**; **Supplementary Figure 3A, B**). In contrast to the data indicating that IL-1β did not induce detectable NO in uninfected Vero E6 cells as measured by flow cytometry (**Supplementary Figure 2A, C**), the increased NO production in SARS-CoV-2 infected, IFN-γ, IL-1β, or IFN-γ plus IL-1β treated cells was further confirmed by flow cytometry (**Figures 2C–E**; **Supplementary Figure 3C**). Furthermore, we found that the dose-response curve for IFN-γ and IL-1β administered together was very close to the sum of the two individual dose-response curves, indicating that the cytokines activate NO production in an additive but not synergistic manner (**Figure 2E**).

The production of NO in Vero E6 cells is regulated by the inducible isoform of the nitric oxide synthase (iNOS) (20), such that we next determined whether the cytokine-mediated induction of NO resulted in increased iNOS expression in infected cells. Vero E6 cells were pretreated with IFN-γ and/or IL-1β, then infected with SARS-CoV-2-mNG and stained with iNOS antibodies at 24 h post-infection for detection by laser scanning confocal microscopy. The mNG fluorescence signal was significantly reduced in cytokine treated cells as compared to media, but less so in cells treated with IL-1β alone as compared with IFN-γ alone or in combination with IL-1β (**Figures 2F, G**). Similarly, Vero E6 cells treated IL-1β alone had significantly lower iNOS expression as compared with IFN-γ alone or in combination with IL-1β (**Figures 2F, G**). Furthermore, colocalization between SARS-CoV-2-mNG and iNOS was only observed in the presence of IFN-γ (**Figures 2F, G**). Taken together, our results demonstrate that stimulation with IFN-γ or IFN-γ plus IL-1β enhances NO production and reduces replication of SARS-CoV-2 in Vero E6 cells.

### IFN-γ-induced killing of SARS-CoV-2 in Vero E6 cells is mediated by nitric oxide

To further evaluate the relationship between NO production and viral replication, we added the pharmacologic inhibitor L-NIL, which is frequently used for these types of studies as it inhibits iNOS activity more efficiently than either of the constitutive endothelial (eNOS or *NOS3*) or neuronal (nNOS or *NOS1*) NO synthases (29, 30, 32). We also studied L-NAME which has a broad spectrum of activity against NO production (29, 31, 46). The addition of L-NIL or L-NAME, but not the inactive enantiomer D-NAME, to IFN-γ plus IL-1β treated Vero E6 cells resulted in decreased DAF-FM positivity, indicating that these inhibitors efficiently blocked NO production in SARS-CoV-2 infected cells (**Supplementary Figure 3D**).

**Figure 3:**
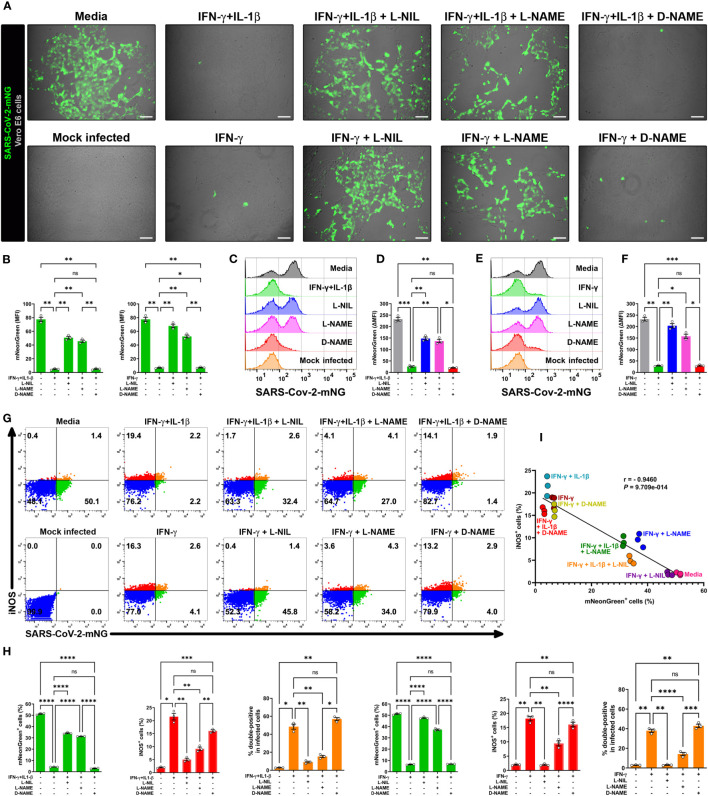
IFN-γ-triggered killing of SARS-CoV-2 in Vero E6 cells is nitric oxide dependent. **(A, B)** Representative live-cell imaging analysis of Vero E6 cells pretreated with IFN-γ (100 U/mL) with or without IL-1β (2.5 ng/mL) and iNOS inhibitors (1 mM L-NIL, 2 mM L-NAME, and 2 mM D-NAME) for 48 h, and infected with SARS-CoV-2-mNG (MOI = 0.1). Scale bars, 100 µm. The MFI of SARS-CoV-2-mNG (green) in Vero E6 cells was quantified with ImageJ software. Data are means ± SEM (*n* = 3). Data were analyzed by one-way ANOVA followed by Tukey’s *post hoc* test (*P < 0.05 and **P < 0.01; ns, not statistically significant). **(C–F)** Flow cytometry analysis of SARS-CoV-2-mNG infected Vero E6 cells treated as described above. FlowJo software was used to determine the ▵MFI of SARS-CoV-2-mNG in Vero E6 cells (▵MFI = MFI_infected_ – MFI_mock infected_). Data are means ± SEM (*n* = 3). Data were analyzed by one-way ANOVA followed by Tukey’s *post hoc* test (*P < 0.05, **P < 0.01, and ***P < 0.001; ns, not statistically significant). **(G–I)** Flow cytometry analysis of SARS-CoV-2-mNG infected Vero E6 cells treated as described above and stained with anti-iNOS Ab conjugated to AF594. **(H)** FlowJo software was used to determine the % of SARS-CoV-2-mNG^+^ (green bars), iNOS^+^ (red bars), and double-positive Vero E6 cells (orange bars). Data are means ± SEM (*n* = 3). Data were analyzed by one-way ANOVA followed by Tukey’s *post hoc* test (*P < 0.05, **P < 0.01, ***P < 0.001, and ****P < 0.0001; ns, not statistically significant). **(I)** Scatter plot showing the inverse relationship between SARS-CoV-2-mNG infection and iNOS expression in cytokine-treated Vero E6 cells. Pearson correlation coefficient (r) and *P* value are indicated in the graph.

We next determined the role of NO production on cytokine-triggered antiviral activity in infected Vero E6 cells by live-cell imaging using the SARS-CoV-2-mNG fluorescent clone. As previously, IFN-γ treatment with or without IL-1β decreased the mNG fluorescence signal in infected cells, but the mNG fluorescence signal was almost completely restored in the presence of L-NIL or L-NAME, but not D-NAME (**Figures 3A, B**). The dependence of NO on IFN-γ-induced antiviral activity with (**Figures 3C, D**) or without IL-1β (**Figures 3E, F**) was further confirmed by flow cytometry. We also found that L-NIL or L-NAME, but not D-NAME inhibited cytokine-induced iNOS protein expression in infected cells as measured by intracellular flow cytometry (**Figures 3G, H**; **Supplementary Figure 3E**), consistent with the known action of these inhibitors (47, 48). Although it was not possible to use live microscopy to measure NO production and antiviral activity in the same cells as the fluorophores had overlapping emission spectra, we were able to simultaneously measure iNOS expression, and antiviral activity as these assays utilize fluorophores with distinct emission spectra (**Figures 3G, H**; **Supplementary Figure 3E**). We observed a significant negative correlation (Pearson’s r = -0.9460) between SARS-CoV-2-mNG intracellular levels and iNOS expression in IFN-γ treated cells. Visualization of scatter diagram revealed that infected Vero E6 cells treated with IFN-γ with or without IL-1β and D-NAME clustered together at the Y-axis with high levels of iNOS and low virus positivity, while untreated media control and cells treated with the cytokines in the presence of iNOS inhibitors L-NIL or L-NAME clustered at the X-axis with high viral levels and inverse iNOS expression (**Figure 3I**). Overall, these data demonstrate the requirement for the induction of NO in IFN-γ-mediated antiviral responses against SARS-CoV-2 in Vero E6 cells.

### Sensitivity of human lung epithelial cells to IFN-γ-induced nitric oxide

In addition to type II IFN (IFN-γ), type I IFNs (IFN-α and IFN-β) and type III IFN (IFN-Λ) also inhibited SARS-CoV-2 replication in lung epithelial cells (18, 28). We therefore sought to compare the ability of all types of IFN to induce NO in pulmonary epithelial cells. We used the human lung epithelial cell line A549-ACE2 which was engineered to stably express the ACE2 receptor (23), thereby facilitating *in vitro* infection by SARS-CoV-2. Again, we measured NO by DAF-FM staining by flow cytometry.

**Figure 4:**
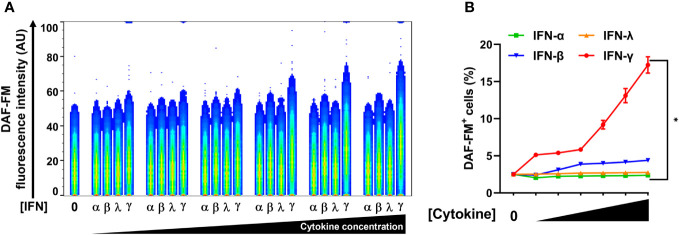
IFN-γ is a strong nitric oxide inducer in human lung epithelial A549-ACE2 cells. **(A, B)** Representative flow cytometry analysis of uninfected A549-ACE2 cells treated with increasing concentrations of IFN-α (10, 50, 100, 200, 400, and 1000 U/mL), IFN-β (10, 50, 100, 200, 400, and 1000 U/mL), IFN-λ (0.1, 0.5, 1, 2, 4, and 10 ng/mL), or IFN-γ (10, 50, 100, 200, 400, and 1000 U/mL) for 48 h, and labeled with nitric oxide indicator DAF-FM. **(A)** Flow cytometry data shown are concatenated FCS files of uninfected A549-ACE2 cells representing the treatment with increasing concentrations of each IFN type. AU, arbitrary units. **(B)** FlowJo software was used to determine the % of uninfected A549-ACE2 cells positive for DAF-FM. 0 indicates media control. Data are means ± SEM (*n* = 3). Data were analyzed by two-way ANOVA followed by Tukey’s *post hoc* test (*P < 0.05). All IFN-γ concentrations were significantly different from the media control. No significant differences were found between the other cytokines and media control except IFN-β at the highest concentration.

While type II/IFN-γ treatment induced NO production in A549-ACE2 cells in a dose-dependent manner, type I/IFN-α and type III/IFN-λ treatments both failed to induce NO (**Figures 4A, B**; **Supplementary Figure 4**). Type I/IFN-β induced NO production only at the highest concentration tested, however, the response for type II/IFN-γ at the same concentration was approximately 4-fold greater (**Figures 4A, B**; **Supplementary Figure 4**). Our data of the IFNs, IFN-γ was the only potent inducer of NO production by human lung epithelial cells.

### Nitric oxide mediates IFN-γ-induced control of SARS-CoV-2 infection in human lung epithelial cells

**Figure 5:**
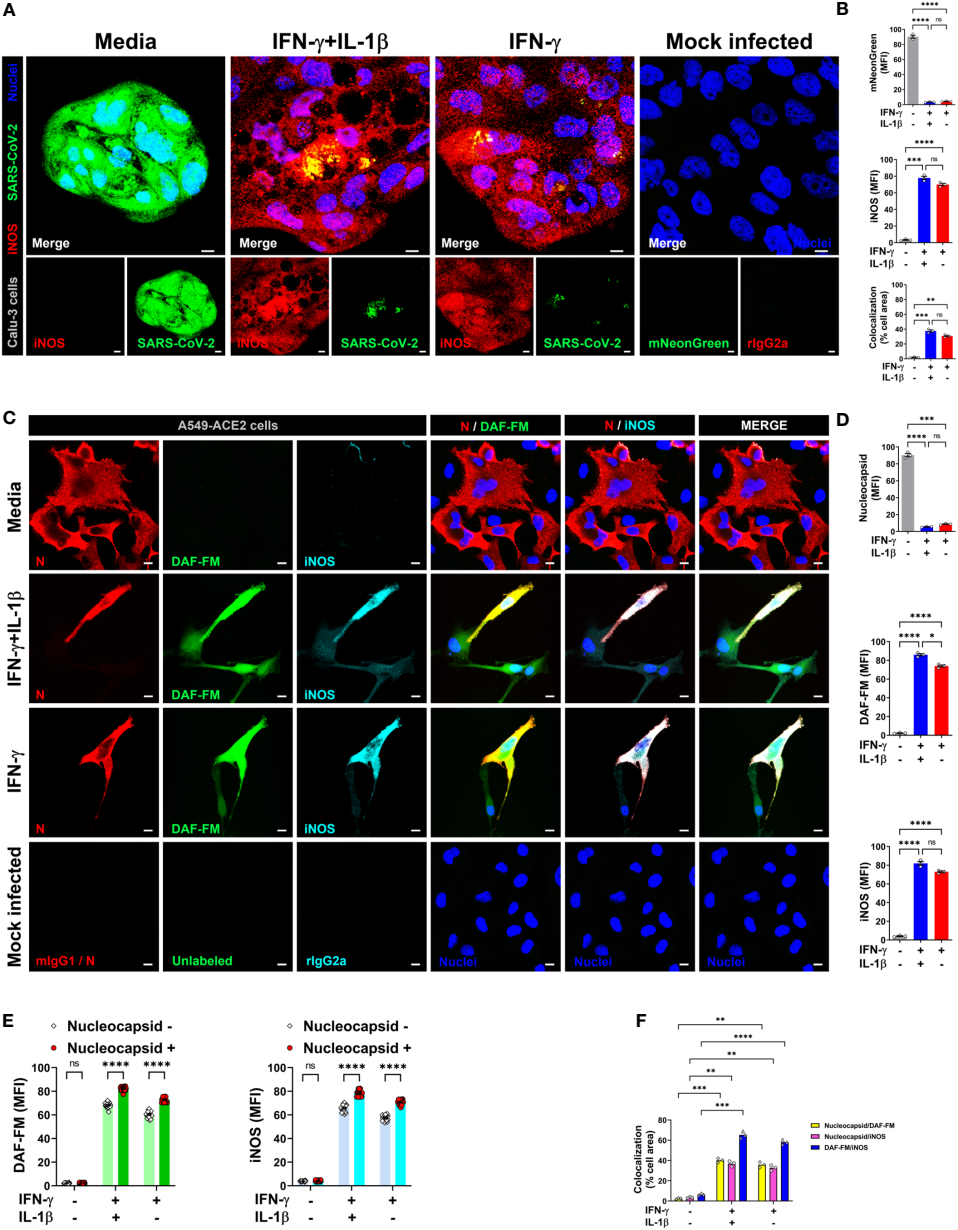
IFN-γ activates nitric oxide pathway in human lung epithelial cells infected with SARS-CoV-2. **(A, B)** Calu-3 cells were pretreated with IFN-γ (400 U/mL) with or without IL-1β (10 ng/mL) for 48 h, mock infected or infected with SARS-CoV-2-mNG (green; MOI = 1), and then stained with anti-iNOS (red) Ab or isotype control and observed by fluorescent confocal microscopy **(A)**. Yellow denotes colocalization between green and red channels. Scale bars, 10 µm. The MFI of SARS-CoV-2-mNG (top), iNOS (middle), and the two-color colocalization (bottom) in infected Calu-3 cells was quantified with ImageJ software **(B)**. Data are means ± SEM (*n* = 3). Data were analyzed by one-way ANOVA followed by Tukey’s *post hoc* test (**P < 0.01, ***P < 0.001, and ****P < 0.0001; ns, not statistically significant). **(C–F)** Representative confocal images of SARS-CoV-2 infected (MOI = 1) A549-ACE2 cells treated as described above and stained with anti-SARS-CoV-2 nucleocapsid Ab (N; red), DAF-FM (green), and anti-iNOS Ab (cyan), or matching isotype controls **(C)**. Nuclei (blue) were counter-stained with NucBlue. Two or all channels (merge) colocalization profiles are shown. Scale bars, 10 µm. The MFI of SARS-CoV-2 nucleocapsid protein, DAF-FM, iNOS, and the two-color colocalization in infected A549-ACE2 cells was quantified with ImageJ software **(D–F)**. Data are means ± SEM (*n* = 3). 20 cells were scored for the nucleoprotein positive versus negative comparison **(E)**. Data were analyzed by one-way ANOVA followed by Tukey’s *post hoc* test (*P < 0.05, **P < 0.01, ***P < 0.001, and ****P < 0.0001; ns, not statistically significant).

Based on our findings that NO drives the antiviral effect downstream of IFN-γ in the simian cell line Vero E6, and the selectivity of A549-ACE2 cells to produce NO toward IFN-γ treatment, we sought to determine whether this cytokine could induce NO-mediated antiviral activity in human lung epithelial cells. To do so, we used two epithelial cell lines derived from human pulmonary adenocarcinomas, Calu-3 and A549-ACE2, which are permissive to SARS-CoV-2 and mimic key features of the human primary pulmonary epithelial cells making them useful for *in vitro* models of infection (28, 49–52). Calu-3 and A549-ACE2 cells were pretreated with cytokines, then infected (MOI = 1) with SARS-CoV-2 wild-type or SARS-CoV-2 expressing mNG fluorescent protein and then labeled with antibodies against iNOS and SARS-CoV-2 nucleocapsid protein. As measured by confocal microscopy, treatment of infected cells with IFN-γ alone or in combination with IL-1β showed greater iNOS expression than media control in both Calu-3 (**Figures 5A, B**; **Supplementary Figure 5A, B**) and A549-ACE2 (**Supplementary Figure 5C–F**) cells. No significant changes were observed in iNOS expression between IFN-γ treatment with or without IL-1β (**Figures 5A, B**; **Supplementary Figure 5A–F**). Conversely, Calu-3 or A549-ACE2 cells incubated with media had higher mNG (**Figures 5A, B**; **Supplementary Figure 5C, D**) or nucleocapsid (**Supplementary Figure 5A, B, E, F**) fluorescence levels than IFN-γ or IFN-γ plus IL-1β treated cells, with the cytokine combination showing the lower positivity for the virus (**Figures 5A, B**; **Supplementary Figure 5A–F**). Furthermore, IFN-γ in combination with IL-1β, or when added alone to the cultures, but not the media control, induced colocalization between SARS-CoV-2-mNG or SARS-CoV-2 nucleocapsid protein and iNOS in both Calu-3 (**Figures 5A, B**; **Supplementary Figure 5A, B**) and A549-ACE2 (**Supplementary Figure 5C–F**) cells.

**Figure 6:**
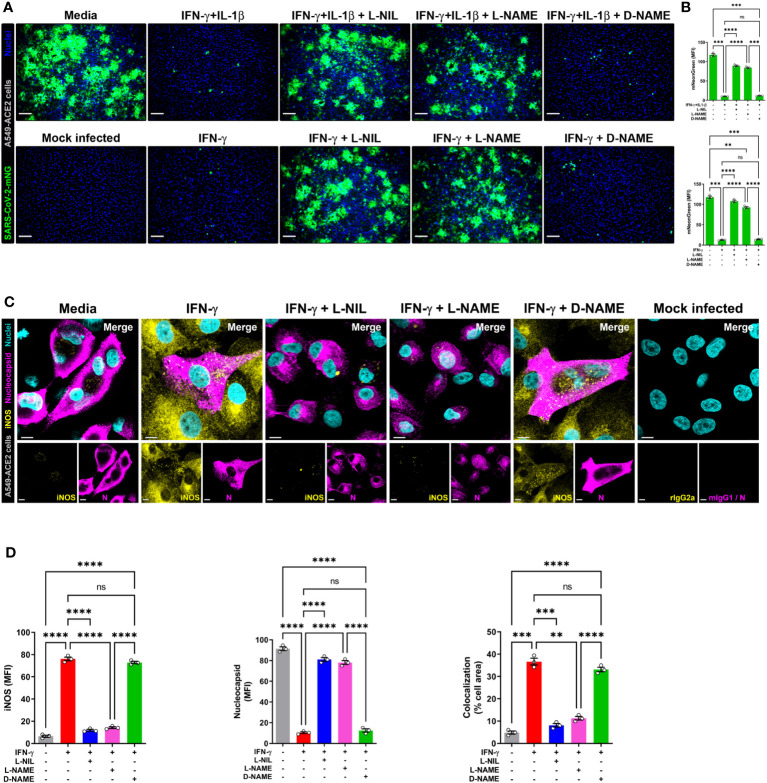
IFN-γ-induced control of SARS-CoV-2 infection in human lung epithelial cells is mediated by nitric oxide. **(A, B)** Representative live-cell imaging analysis of A549-ACE2 cells pretreated with IFN-γ (100 U/mL) with or without IL-1β (2.5 ng/mL) and iNOS inhibitors (1 mM L-NIL, 2 mM L-NAME, and 2 mM D-NAME) for 48 h, and infected with SARS-CoV-2-mNG (green; MOI = 1). Nuclei (blue) were counter-stained with NucBlue. Scale bars, 100 µm. The MFI of SARS-CoV-2-mNG in A549-ACE2 cells was quantified with ImageJ software. Data are means ± SEM (*n* = 3). Data were analyzed by one-way ANOVA followed by Tukey’s *post hoc* test (**P < 0.01, ***P < 0.001, and ****P < 0.0001; ns, not statistically significant). **(C, D)** Representative confocal images of SARS-CoV-2 infected (MOI = 1) A549-ACE2 cells treated with IFN-γ as described above and stained with anti-SARS-CoV-2 nucleocapsid Ab (N; magenta) and anti-iNOS Ab (yellow), or matching isotype controls **(C)**. Nuclei (cyan) were counter-stained with NucBlue. Scale bars, 10 µm. The MFI of SARS-CoV-2 nucleocapsid protein, iNOS, and the two-color colocalization in infected A549-ACE2 cells was quantified with ImageJ software **(D)**. Data are means ± SEM (*n* = 3). Data were analyzed by one-way ANOVA followed by Tukey’s *post hoc* test (**P < 0.01, ***P < 0.001, and ****P < 0.0001; ns, not statistically significant).

**Figure 7:**
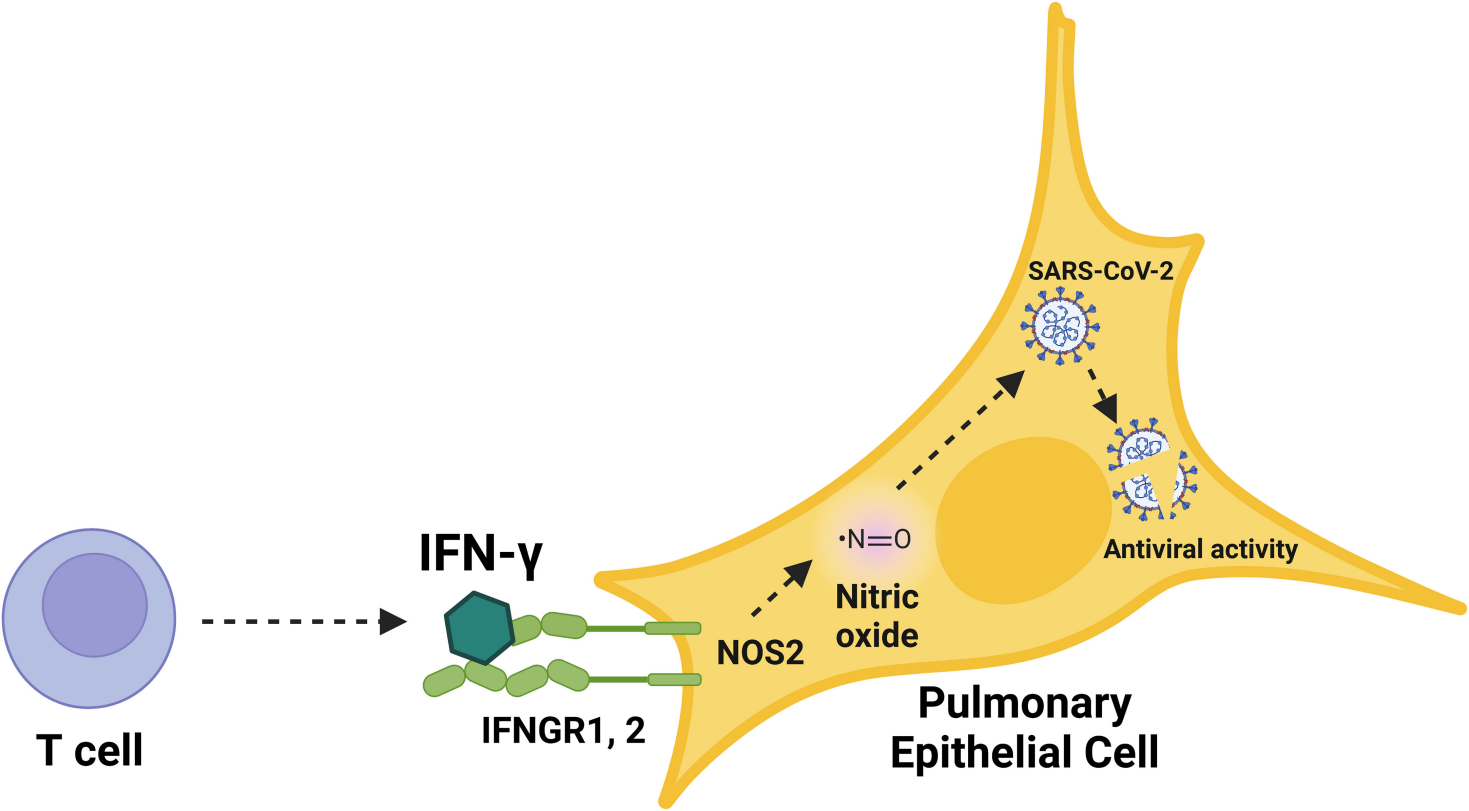
Schematic overview of the immunological induction of nitric oxide by IFN-γ to control SARS-CoV-2 infection in human pulmonary epithelial cells. Graphical summary of nitric oxide-mediated, cytokine-triggered anti-SARS-CoV-2 viral activity in lung epithelial cells. Our data showed that IFN-γ, which is mainly produced by CD8^+^ T cells in response to SARS-CoV-2 infection, interfered with viral activity through the induction of iNOS/NOS2 and subsequent production of nitric oxide. The inhibition of SARS-CoV-2 replication cycle induced by IFN-γ was prevented by the treatment with iNOS/NOS2 inhibitors, demonstrating the requirement for nitric oxide in IFN-γ-mediated antiviral responses in human cells.

Next, to detect NO and SARS-CoV-2 in the same cells, we overcame the spectral overlap issue of mNG and DAF-FM by infecting A549-ACE2 cells with non-fluorescent SARS-CoV-2 (MOI = 1) followed by DAF-FM staining and virus detection by anti-SARS-CoV-2 nucleocapsid antibody and immediately imaged the cells by confocal microscopy. IFN-γ treatment with or without IL-1β reduced the number of SARS-CoV-2 infected A549-ACE2 cells in comparison to media control, whereas greater DAF-FM staining was observed in cells treated with IFN-γ plus IL-1β than untreated or cells singly treated with IFN-γ (**Figures 5C, D**; **Supplementary Figure 6**). By comparing the MFI of DAF-FM and iNOS in cells positive or negative for SARS-CoV-2 nucleocapsid protein, we found that stimulation with IFN-γ or IFN-γ plus IL-1β but not media control induced more NO production in infected versus uninfected cells (**Figures 5C, E**; **Supplementary Figure 6**). Additionally, a stronger colocalization between SARS-CoV-2 nucleocapsid and DAF-FM was observed in cytokine treated as compared to media treated cells (**Figures 5C, F**; **Supplementary Figure 6**), indicating that IFN-γ targets the virus to the NO pathway for destruction. We also demonstrated that SARS-CoV-2 nucleoprotein colocalizes with both DAF-FM and iNOS in cytokine-stimulated but not media control A549-ACE2 cells (**Figures 5C, F**; **Supplementary Figure 6**). In IFN-γ treated cells, with or without IL-1β, no significant differences were observed on the colocalization between SARS-CoV-2 nucleoprotein and DAF-FM or iNOS (**Figures 5C, F**; **Supplementary Figure 6**). Finally, we determined the role of NO production on cytokine-triggered antiviral activity in human cells by adding pharmacologic inhibitors of iNOS to the SARS-CoV-2-mNG infected cultures. As measured by live-cell imaging, IFN-γ treatment with or without IL-1β decreased the mNG fluorescence signal in infected A549-ACE2 cells, with no significant differences observed when IFN-γ was added in combination with IL-1β or not (**Figures 6A, B**) as previously shown. The addition of the iNOS inhibitors L-NIL or L-NAME, but not the inactive enantiomer D-NAME, to IFN-γ or IFN-γ plus IL-1β treated A549-ACE2 cells resulted in an increase of mNG fluorescence signal (**Figures 6A, B**). Furthermore, the addition of the iNOS inhibitors L-NIL or L-NAME, but not D-NAME, to IFN-γ-treated A549-ACE2 cells resulted in increased fluorescence levels of SARS-CoV-2 nucleocapsid, whereas decreased iNOS positivity and colocalization between SARS-CoV-2 nucleoprotein and iNOS were observed by confocal microscopy (**Figures 6C, D**), indicating that these inhibitors efficiently blocked the NO-mediated antiviral effect triggered by IFN-γ. Altogether, these data suggest that NO production is required for the antiviral activity induced by IFN-γ against SARS-CoV-2 in human lung epithelial cells (**Figure 7**).

## Discussion

Identification of the host pathways that combat SARS-CoV-2 infection in humans is key toward developing both preventative and therapeutic strategies to limit the ongoing pandemic. Here, we studied the role of IFN-γ, given that its production coincides with the onset of protective immunity to SARS-CoV-2 post vaccination and its administration to immunocompromised patients results in viral clearance and resolution of symptoms (2, 3, 15). Given the role of IFN-γ in inducing NO, a key biological mediator in the immune system with broad antimicrobial activity against intracellular pathogens (53, 54), we evaluated whether IFN-γ triggers an antimicrobial response using a SARS-CoV-2 reporter virus. We demonstrate that IFN-γ inhibits the SARS-CoV-2 replication cycle in both simian and human epithelial cell lines, finding that the IFN-γ-induced anti-SARS-CoV-2 viral activity is mediated through the endogenous production of NO. IL-1β, which, when combined with IFN-γ is known to have an antiviral effect on the replication cycle of SARS-CoV-1 (20), enhanced IFN-γ induction of NO, but it had little effect on subsequent antiviral activity. These data indicate that IFN-γ, known to be produced early during infection or post-vaccination by CD8^+^ T cells (2, 3), is sufficient to trigger the NO-dependent killing of SARS-CoV-2 in lung epithelial cells.

Our flow cytometry and live microscopy analysis of SARS-CoV-2 infected Vero E6 cells, showed that IFN-γ or a combination of IFN-γ and IL-1β induced the iNOS-dependent production of NO, which resulted the inhibition of SARS-CoV-2 replication. The antiviral activity induced by IFN-γ alone or together with IL-1β was not restricted to the reduction of the percentage of SARS-CoV-2 infected cells but extended to a reduced intracellular virus yield and release of extracellular virions. We have not tested whether IFN-γ could block SARS-CoV-2 replication after the viral infection has been already established, future studies might address whether IFN-γ blocks SARS-CoV-2 infection after viral entry. The replication of SARS-CoV-2 in Vero E6 cells was inhibited by the NO donor drug SNAP, which directly releases NO (33). The SNAP-mediated antiviral effect was associated with NO targeting of the SARS-CoV-2 3CL cysteine protease, although the viral replication cycle was not completely blocked (33). Due to limited availability and side effects of NO donors, alternative strategies such as direct administration of NO through gas inhalation have been utilized for potential COVID-19 treatments and clinical trials (54–58). It has been proposed that NO could inhibit the replication of SARS-CoV-2 by decreasing the intracellular calcium levels, which impairs the action of the calcium-dependent protease furin, a host cell protein utilized by SARS-CoV-2 to replicate in the respiratory tract (26, 55). One specific limitation of our study is the lack of a direct or indirect mechanism by which NO restrains SARS-CoV-2 replication cycle. However, we believe it is likely that the NO-mediated antiviral effects are intracellular rather than occurring on the extracellular virions, as has been demonstrated for the antiviral activity induced by IFN-γ and IL-1β in hantavirus-infected cells (42).

It is known that excessive NO levels can induce cell death in many cell types (45, 59, 60). We note that IFN-γ alone or in combination with IL-1β did not induce NO-mediated cell death in uninfected Vero E6 cells. In fact, IFN-γ stimulation protected cells against death, perhaps by inducing pro-survival pathways such as autophagy (39, 40, 61), while the control cells might have undergone growth arrest and activated death signaling pathways due to the lack of stimuli (60). In contrast, IFN-γ in combination with TNF or LPS induced NO and licensed programmed cell death (59, 62), although treatment with any of these agonists individually did not. It is difficult to determine the effect of NO induction in infected cells, as infection itself causes cell death. It might be possible to treat with IFN-γ alone at an optimal dose to augment NO-induced antiviral activity while favoring autophagy vs. apoptosis.

IL-1β is well known to induce antimicrobial responses against virus, bacteria, and protozoa (43, 63, 64) and to enhance the antiviral effect of both IFN-α and IFN-γ (20, 65). However, in our study, the addition of IL-1β to the SARS-CoV-2 infected cultures did not significantly amplify the IFN-γ-induced antiviral activity, although NO production was increased. When exposed to a cytokine mixture of IFN-γ and IL-1β, murine bone marrow-derived macrophages, and simian and human renal epithelial cells, showed an increased nitrite production but exposure to IL-1β alone failed to induce nitrite formation, indicating that the IL-1β-induced NO production in those cells was dependent on the presence of IFN-γ (42, 64, 66). IL-1β induced NO formation in the absence of IFN-γ in human chondrocytes, and rat myocytes and hepatocytes but not Kupffer cells, these differences are likely to reflect cell types and species variations in the regulation of *NOS2* gene promoter (67–70). We found that IFN-γ was the primary trigger for NO production in pulmonary epithelial cells, such that experiments using a suboptimal dose of IFN-γ are likely required to further define the role of IL-1β in the antiviral response to SARS-CoV-2.

Given that type I and type III IFNs have been shown to induce an antiviral activity against SARS-CoV-2 (18, 28), we compared the activity of the IFNs in inducing NO. As opposed to the potent activity of IFN-γ in inducing NO production in the lung epithelial cell line A549-ACE2, the type I IFNs and type III IFN were either unable to induce NO or minimally induced NO production. This is consistent with the finding that type I IFN did not induce *NOS2* mRNA in NHBE cells (71). Previous studies have shown that IFN-α generally increases and IFN-β decreases NO production in human cells, although this was not tested in lung epithelial cells (72, 73). COVID-19 disease initially results in impaired production of type I IFNs (9–12). In addition, type III IFN production is initially impaired in COVID-19 (12), although later IFN-λ secretion upon viral recognition causes damage to the lung epithelial barrier, predisposing the host to lethal bacterial superinfections (74). Although type I and type III IFNs have been shown to have antiviral activity in lung epithelial cells, our data suggests that the mechanism is NO independent. Since type III IFNs signal through a distinct receptor complex that is restricted to epithelial cells, which is also expressed in the lung epithelial cell line A549 (75), it is likely that the different IFNs induce distinct patterns of ISGs (18, 28, 76). We hypothesize that these variations in IFN-induced ISGs contribute to differential levels of NO induction. One such limitation of our study is that we did not verify the expression of IFN receptors on the lung epithelial cells, although these cells are known to respond to the different IFNs (18, 77) and express the distinct IFN receptors (75, 78, 79).

It is likely that *in vivo* T cells are the source of IFN-γ required to activate the NO-dependent SARS-CoV-2 antiviral activity. The frequencies of IFN-γ-producing NK and T cells are significantly decreased in COVID-19 patients, with a near complete reduction of IFN-γ-producing NK cells (80–83). This is consistent with studies indicating the critical importance of T cells in the clearance of SARS-CoV-2 infection and subsequent disease resolution (83–85). In addition to their ability to secrete IFN-γ in response to SARS-CoV-2 peptide antigens (86–88), CD4^+^ T cells recruit and activate multiple cell types, whereas, CD8^+^ T cells (CTLs) are thought to directly contribute to an antiviral response through their cytolytic activity, depleting the reservoir of infected cells. The number of differentiated granulysin (GNLY)^+^CD8^+^ CTLs increases during infection and convalescence (89), yet the exhaustion of CD8^+^ CTLs in COVID-19 disease was associated with the increased expression of the inhibitory receptor NKG2A (80). We previously described a GNLY-expressing CD8^+^ CTL subset expressing NKG2C but exhibited an antimicrobial activity against *Mycobacterium leprae*, but those expressing NKG2A showed a decrease antimicrobial activity (90). GNLY expressing CTLs are the most mature.

Disease severity in COVID-19 is associated with a dysregulated immune response, which includes alterations in both IFN and proinflammatory responses, indicating that the timing and duration of the cytokine response need to be properly regulated (17, 91–93). While most studies focus on the use of type I IFNs for the treatment of COVID-19, very few studies have explored the use of type II IFNs. To date, only one study, which used the lung epithelial cell line Calu-3, has shown that IFN-γ can inhibit SARS-CoV-2 replication in human cells, and only a single clinical trial using IFN-γ has been conducted (18, 94). Treatment of primary lung epithelial cells with IFN-γ inhibited intracellular SARS-CoV-2 replication, albeit to a lesser extent than that observed with type I IFN (18). It is important to acknowledge that the interpretation of these results was limited by the dataset’s scope, encompassing only two donors, and was further complicated by disparities in the experimental conditions relative to those employed for the cell lines (18). Despite these limitations, the present study’s emphasis was placed on highlighting the antiviral activity induced by IFN-γ in pulmonary epithelial cell lines, given the robust effect, facilitating investigating the role of NO in this response. It is essential to recognize that further investigations are warranted to validate this mechanistic pathway in primary cells.

The successful use of IFN-γ on the treatment of five immunocompromised patients with prolonged COVID-19 has been described (15, 17). Although a small cohort, all five patients had SARS-CoV-2 clearance and improvement of respiratory status, and four patients showed clinical recovery with no evidence of hyperinflammation (15, 17). It has been shown that pretreatment with IFN-γ blocks SARS-CoV-2 infection in Calu-3 cells, but the antiviral mechanism was not clear, although it involved a weak ISGs mRNA response and the cell surface upregulation of ACE2 receptor (18). By integrating live-cell and confocal microscopy, it was possible to delineate the anti-SARS-CoV-2 viral activity of IFN-γ in two human lung epithelial cell lines, A549 and Calu-3, using a lower concentration of IFN-γ than had been previously reported to contribute to antiviral defense in Calu-3 cells (18). Although treatment with type III IFN (peginterferon lambda) has been shown to reduce hospitalization and emergency room visits in patients with COVID-19, it did not reduce viral shedding (95, 96). In contrast, administration of high dose IFN-β/type I IFN showed no clinical improvement (97).

Our findings suggest that the NO-dependent, cytokine-triggered antiviral effect identified here may benefit patients with COVID-19 and offer potential therapeutic strategies for immune control of SARS-CoV-2 infection. Collectively, we provide evidence, previously unappreciated, of a mechanism of immunological induction of NO production to control SARS-CoV-2 infection.

## Data availability statement

The original contributions presented in the study are included in the article/**Supplementary Material**. Further inquiries can be directed to the corresponding author.

## Ethics statement

Ethical approval was not required for the studies on humans in accordance with the local legislation and institutional requirements because only commercially available established cell lines were used.

## Author contributions

BA: Conceptualization, Investigation, Methodology, Visualization, Writing – original draft, Writing – review & editing. PK: Investigation, Methodology, Visualization, Writing – review & editing. RT: Investigation, Methodology, Visualization, Writing – review & editing. PA: Investigation, Methodology, Visualization, Writing – review & editing. JR: Investigation, Visualization, Writing – review & editing. MF: Investigation, Visualization, Writing – review & editing. OY: Investigation, Visualization, Writing – review & editing. BB: Investigation, Supervision, Visualization, Writing – review & editing. RM: Conceptualization, Funding acquisition, Investigation, Supervision, Visualization, Writing – original draft, Writing – review & editing.
