# Supplementary material for: IFN-γ-mediated control of SARS-CoV-2 infection through nitric oxide
Source: Front Immunol. 2023 Dec 15;14:1284148. doi: 10.3389/fimmu.2023.1284148 (PMC10755032; doi:10.3389/fimmu.2023.1284148)
Supplement: Supplementary file 1 [file DataSheet_1.docx]

Supplementary Material

## Supplementary Figures

**
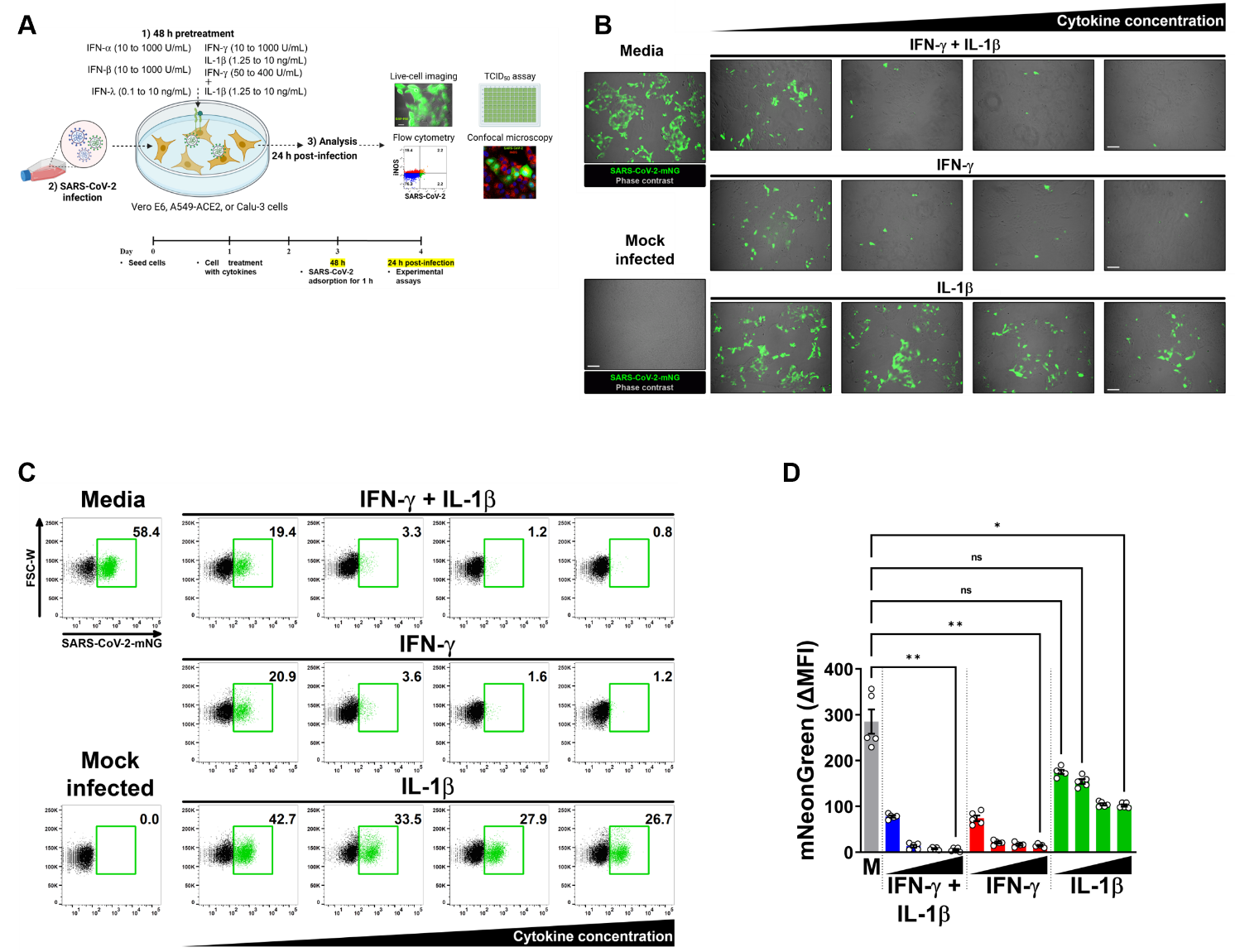
**

**Supplementary Figure 1.** IFN-γ treatment decreases SARS-CoV-2 infection in Vero E6 cells. **(A)** Schematic diagram of cytokine pretreatment, viral infection, and experimental assays. Created with [BioRender.com](https://biorender.com/). **(B)** Representative live-cell imaging analysis of Vero E6 cells pretreated with increasing concentrations of IFN-γ (50, 100, 200, 400 U/mL), IL-1β (1.25, 2.5, 5, or 10 ng/mL), or IFN-γ in combination with IL-1β (50/1.25, 100/2.5, 200/5, or 400/10, respectively) for 48 h, and infected with SARS-CoV-2-mNG (MOI = 0.1). Scale bars, 100 µm. The images are linked to the experiments described in Figure 1A, B. **(C, D)** Flow cytometry analysis of SARS-CoV-2-mNG infected Vero E6 cells treated as described above. **(D)** FlowJo software was used to determine the ΔMFI of SARS-CoV-2-mNG in Vero E6 cells (ΔMFI = MFI_infected_ – MFI_mock infected_). M, media. Data are means ± SEM (*n* = 5). Data were analyzed by one-way ANOVA followed by Tukey’s post hoc test (**P* < 0.05 and ***P* < 0.01; ns, not statistically significant). All cytokine concentrations except IL-1β at the two lowest concentrations were significantly different from the media control. The data are linked to the experiments described in Figure 1C–E.

**
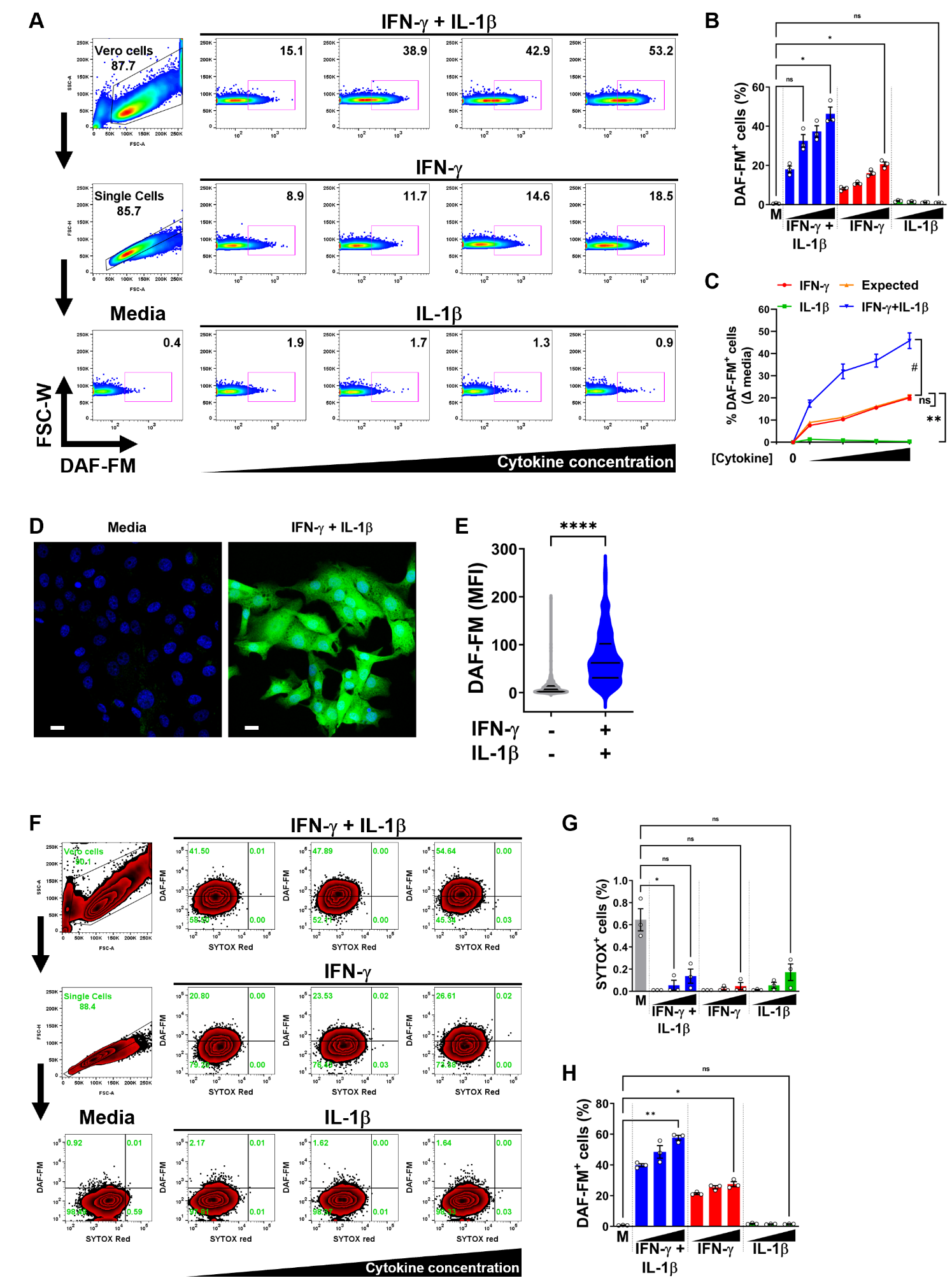
**

**Supplementary Figure 2.** IFN-γ induces nitric oxide production but not cell death in Vero E6 cells. **(A–C)** Representative flow cytometry analysis of uninfected Vero E6 cells treated with increasing concentrations of IFN-γ (50, 100, 200, 400 U/mL), IL-1β (1.25, 2.5, 5, or 10 ng/mL), or IFN-γ in combination with IL-1β (50/1.25, 100/2.5, 200/5, or 400/10, respectively) for 48 h, and labeled with nitric oxide indicator DAF-FM (A). **(B)** FlowJo software was used to determine the % of uninfected Vero E6 cells positive for DAF-FM. M, media. Data are means ± SEM (*n* = 3). Data were analyzed by one-way ANOVA followed by Tukey’s post hoc test (**P* < 0.05; ns, not statistically significant). All IFN-γ concentrations (together or not with IL-1β) were significantly different from the media control except IFN-γ in combination with IL-1β at the second lowest concentration. No significant differences were observed between the IL-1β treatment and media control. **(C)** Dose-response curves of DAF-FM signal induced by cytokines treatment. Data are means ± SEM (*n* = 3). Data were analyzed by two-way ANOVA followed by Tukey’s post hoc test (^#^ *P* < 0.05 and ***P* < 0.01; ns, not statistically significant). **(D)** Representative confocal images of uninfected Vero E6 cells treated with IFN-γ (400 U/ml) and IL-1β (10 ng/mL) for 48 h and labeled with DAF-FM. Scale bars, 50 µm. **(E)** The MFI of DAF-FM in Vero E6 cells was quantified with ImageJ software. Violin plots show the median and quartiles (*n* = 3). Data were analyzed by unpaired Mann-Whitney test (*****P* < 0.0001). **(F–H)** Representative flow cytometry analysis of uninfected Vero E6 cells treated with IFN-γ (400, 800, and 1600 U/mL), IL-1β (10, 20, and 40 ng/mL), or cytokine combinations of IFN-γ and IL-1β (400/10, 800/20, and 1600/40, respectively) for 48 h, and double stained with SYTOX Red dead-cell indicator and DAF-FM diacetate. **(G)** FlowJo software was used to determine the % of total SYTOX^+^ Vero E6 cells. M, media. Data are means ± SEM (*n* = 3). Data were analyzed by one-way ANOVA followed by Tukey’s post hoc test (**P* < 0.05; ns, not statistically significant). No significant differences were observed between the IFN-γ treatment (with or without IL-1β) and media control except IFN-γ in combination with IL-1β at the intermediate concentration (800/20). **(H)** FlowJo software was used to determine the % of live DAF-FM^+^ Vero E6 cells. M, media. Data are means ± SEM (*n* = 3). Data were analyzed by one-way ANOVA followed by Tukey’s post hoc test (**P* < 0.05 and ***P* < 0.01; ns, not statistically significant). All IFN-γ concentrations (together or not with IL-1β) were significantly different from the media control. No significant differences were observed between the IL-1β treatment and media control.


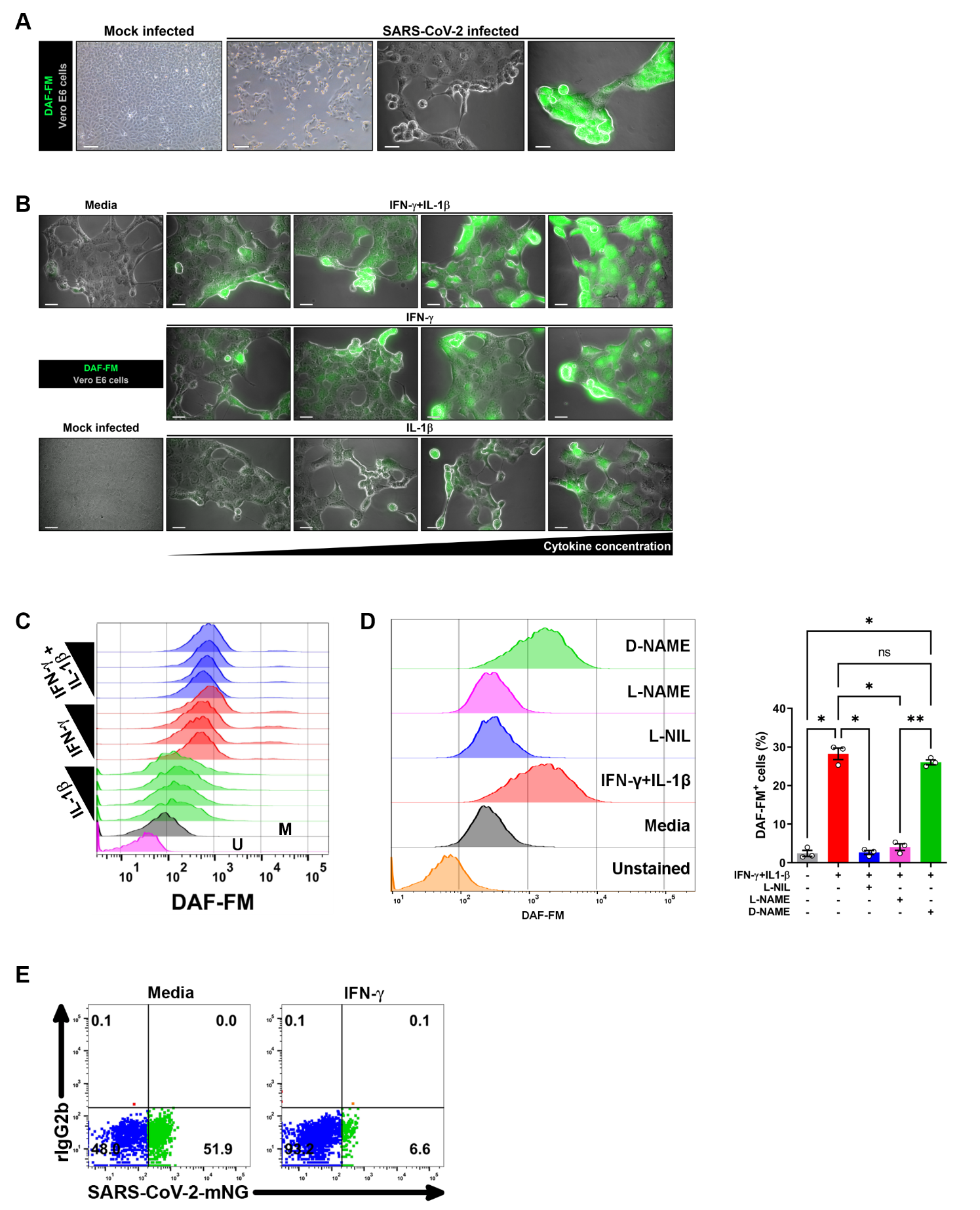


**Supplementary Figure 3.** IFN-γ induces nitric oxide production in SARS-CoV-2-infected Vero E6 cells. **(A)** Representative images of the CPE induced by SARS-CoV-2 infection (MOI = 0.1) in Vero E6 cells. IFN-γ (400 U/mL) plus IL-1β (10 ng/mL) treated cells (48 h) displaying CPE and positivity for DAF-FM (green) are shown (right). Scale bars, 50 µm. **(B)** Representative live-cell imaging analysis of Vero E6 cells pretreated with increasing concentrations of IFN-γ (50, 100, 200, 400 U/mL), IL-1β (1.25, 2.5, 5, or 10 ng/mL), or IFN-γ in combination with IL-1β (50/1.25, 100/2.5, 200/5, or 400/10, respectively) for 48 h, infected with SARS-CoV-2 (MOI = 0.1), and labeled with DAF-FM (green). Scale bars, 50 µm. The images are linked to the experiments described in Figure 2A, B. **(C)** Flow cytometry analysis of DAF-FM. Infected Vero E6 cells were treated and stained as described in (B). U, unstained; M, media. The data are linked to the experiments described in Figure 2C–E. **(D)** Representative flow cytometry analysis of Vero E6 cells pretreated with IFN-γ plus IL-1β for 48 h at the highest concentrations (400 U/ml and 10 ng/mL, respectively) in the presence or absence of iNOS inhibitors (1 mM L-NIL, 2 mM L-NAME, and 2 mM D-NAME), infected with SARS-CoV-2 (MOI = 0.1), and labeled with DAF-FM. FlowJo software was used to determine the % of infected Vero E6 cells positive for DAF-FM. Data are means ± SEM (*n* = 3). Data were analyzed by one-way ANOVA followed by Tukey’s post hoc test (**P* < 0.05 and ***P* < 0.01; ns, not statistically significant). **(E)** Flow cytometry analysis of SARS-CoV-2-mNG infected (MOI = 0.1) Vero E6 cells stained with rat IgG2bκ isotype control Ab conjugated to AF594. Representative untreated (media) and treated (IFN-γ) samples are shown (48 h treatment). FlowJo software was used to determine the % of positive cells displayed in the dot plots. The data are linked to the experiments described in Figure 3G–I.

**
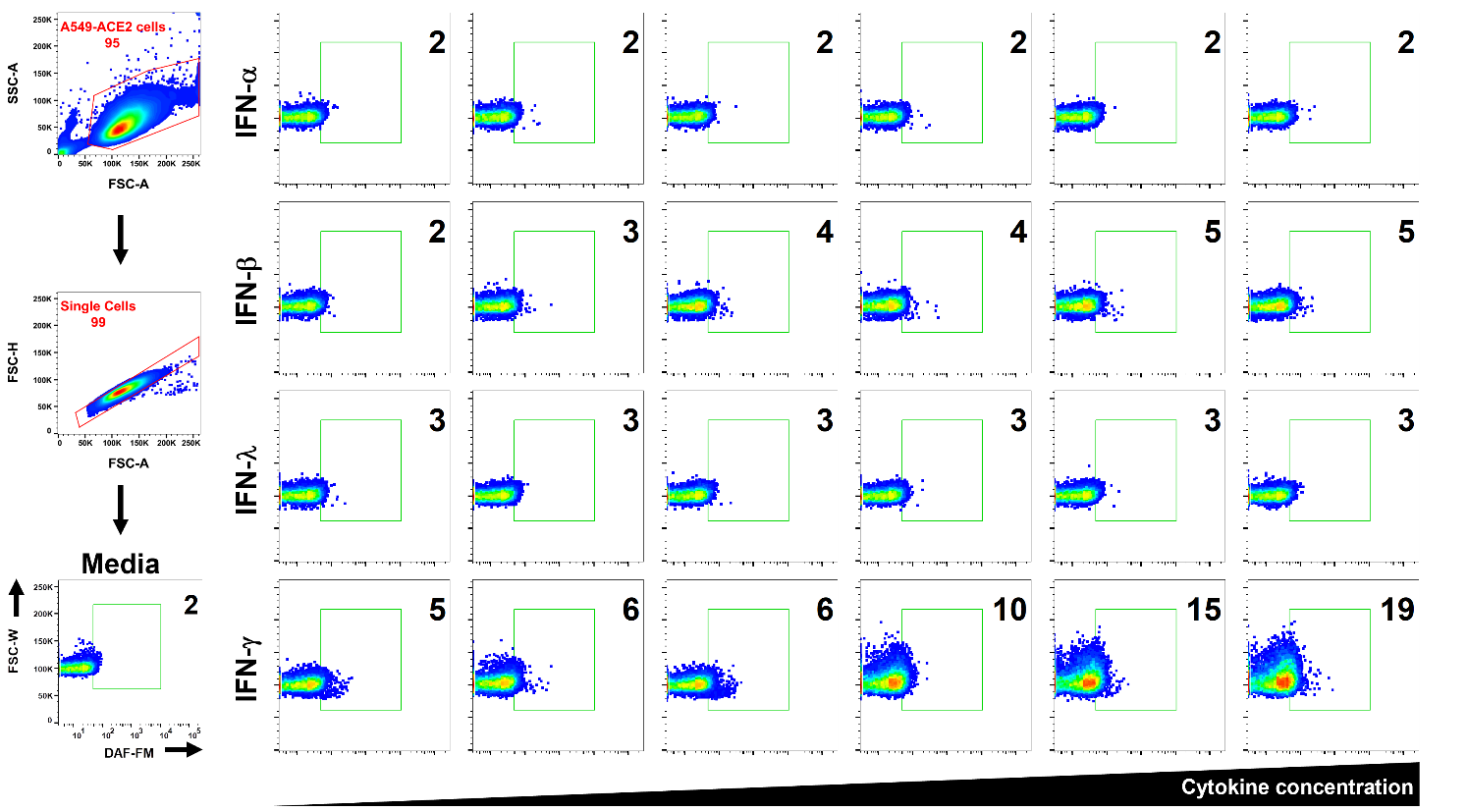
**

**Supplementary Figure 4.** IFN-γ is a potent nitric oxide inducer in human lung epithelial cells. Representative flow cytometry analysis of uninfected A549-ACE2 cells treated with increasing concentrations of IFN-α (10, 50, 100, 200, 400, and 1000 U/mL), IFN-β (10, 50, 100, 200, 400, and 1000 U/mL), IFN-λ (0.1, 0.5, 1, 2, 4, and 10 ng/mL), or IFN-γ (10, 50, 100, 200, 400, and 1000 U/mL) for 48 h and labeled with nitric oxide indicator DAF-FM. FlowJo software was used to determine the % of uninfected A549-ACE2 cells positive for DAF-FM. Dot plots are representative of three independent experiments. The data are linked to the experiments described in Figure 4A, B.


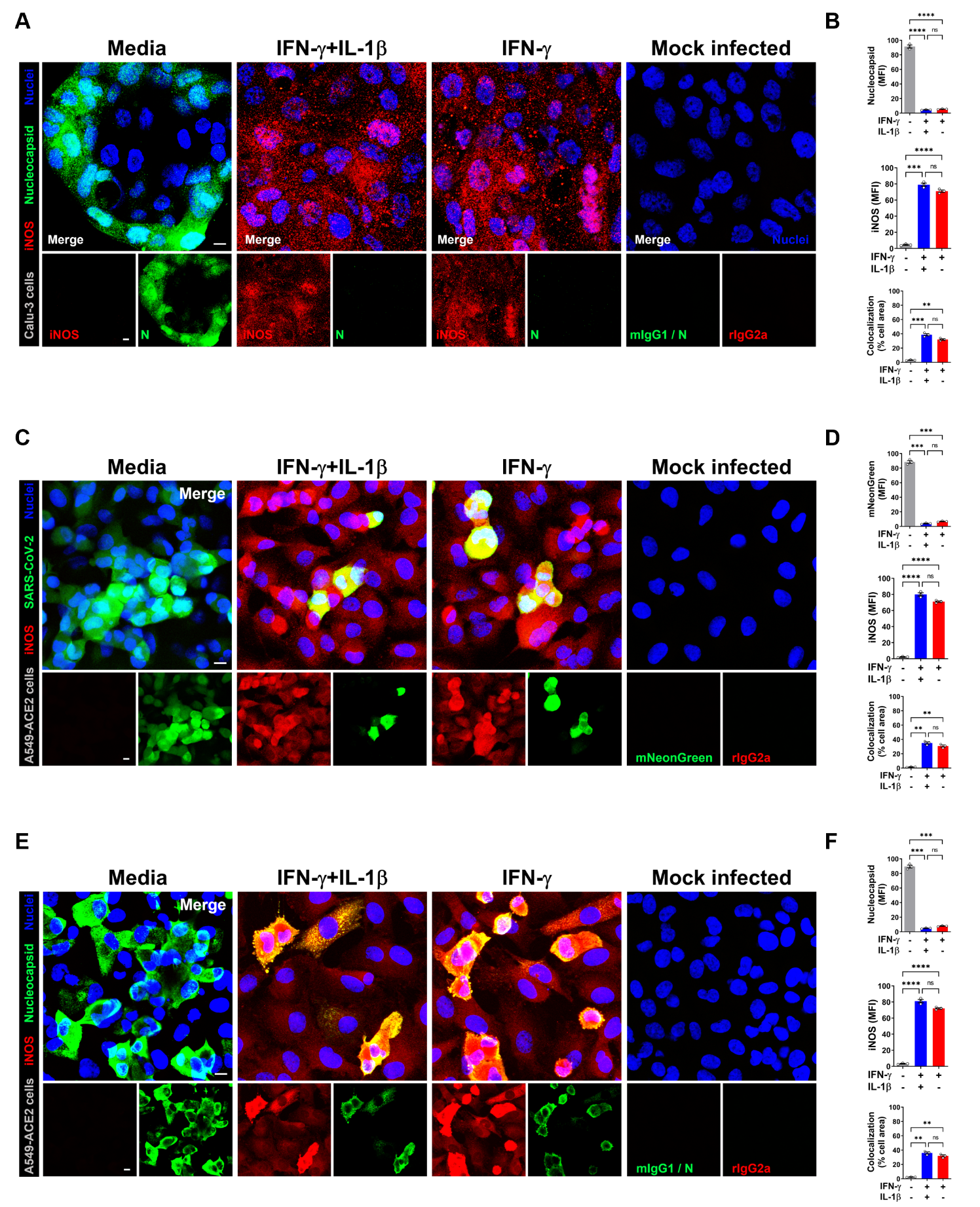


**Supplementary Figure 5.** IFN-γ treatment triggers nitric oxide pathway in human lung epithelial cells infected with SARS-CoV-2. **(A)** Representative confocal images of Calu-3 cells pretreated with IFN-γ with or without IL-1β for 48 h at the highest concentrations (400 U/ml and 10 ng/mL, respectively), mock-infected or infected with SARS-CoV-2 (MOI = 1), and then stained with anti-SARS-CoV-2 nucleocapsid Ab (N; green), anti-iNOS Ab (red), or matching isotype controls. Nuclei (blue) were counter-stained with NucBlue. Yellow denotes colocalization between green and red channels. Scale bars, 10 µm. **(B)** The MFI of SARS-CoV-2 nucleocapsid protein (top), iNOS (middle), and the two-color colocalization (bottom) in infected Calu-3 cells was quantified with ImageJ software. Data are means ± SEM (*n* = 3). Data were analyzed by one-way ANOVA followed by Tukey’s post hoc test (***P* < 0.01, ****P* < 0.001, and *****P* < 0.0001; ns, not statistically significant). **(C)** Representative confocal images of SARS-CoV-2-mNG infected (green; MOI = 1) A549-ACE2 cells treated as described above and then stained with anti-iNOS (red) Ab or isotype control. Nuclei (blue) were counter-stained with NucBlue. Yellow denotes colocalization between green and red channels. Scale bars, 10 µm. **(D)** The MFI of SARS-CoV-2-mNG (top), iNOS (middle), and the two-color colocalization (bottom) in infected A549-ACE2 cells were quantified with ImageJ software. Data are means ± SEM (*n* = 3). Data were analyzed by one-way ANOVA followed by Tukey’s post hoc test (***P* < 0.01, ****P* < 0.001, and *****P* < 0.0001; ns, not statistically significant). **(E)** Representative confocal images of SARS-CoV-2 infected (MOI = 1) A549-ACE2 cells treated as described in (A), and then stained with anti-SARS-CoV-2 nucleocapsid Ab (N; green), anti-iNOS Ab (red), or matching isotype controls. Nuclei (blue) were counter-stained with NucBlue. Yellow denotes colocalization between green and red channels. Scale bars, 10 µm. **(F)** The MFI of SARS-CoV-2 nucleocapsid protein (top), iNOS (middle), and the two-color colocalization (bottom) in infected A549-ACE2 cells were quantified with ImageJ software. Data are means ± SEM (*n* = 3). Data were analyzed by one-way ANOVA followed by Tukey’s post hoc test (***P* < 0.01, ****P* < 0.001, and *****P* < 0.0001; ns, not statistically significant).

**
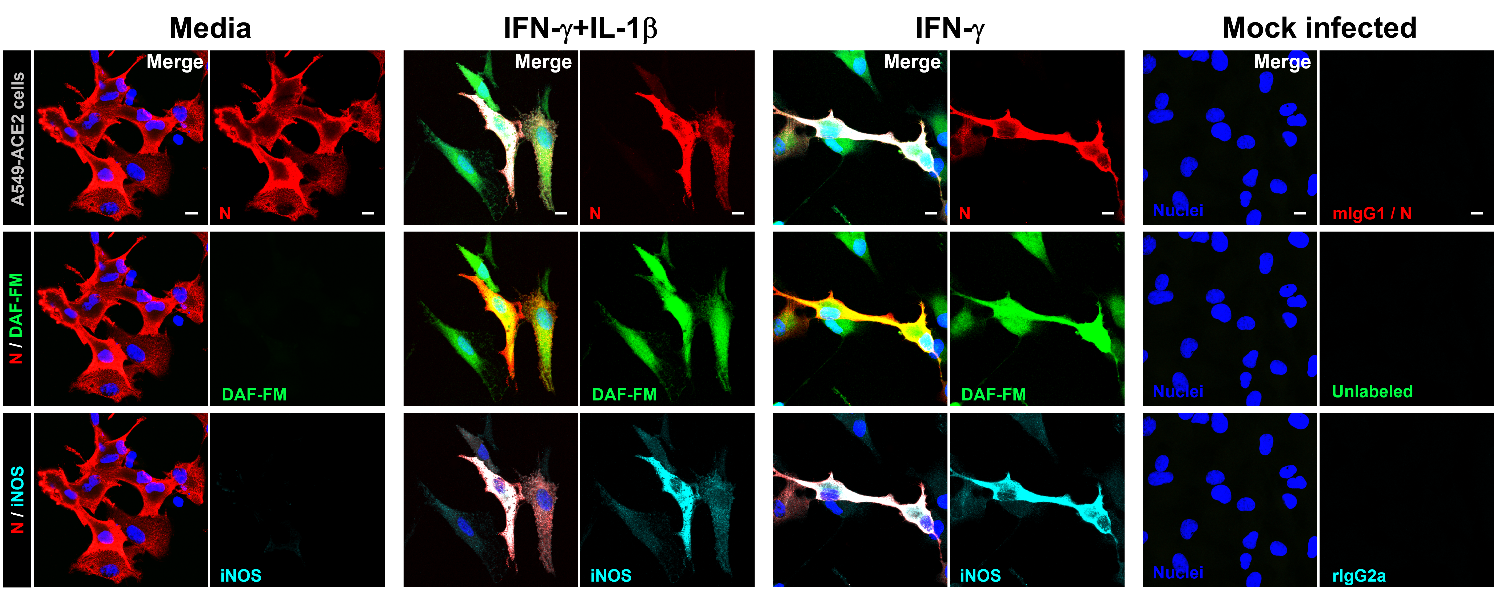
**

**Supplementary Figure 6.** IFN-γ treatment activates nitric oxide pathway in A549-ACE2 cells infected with SARS-CoV-2. Representative confocal images of SARS-CoV-2 infected (MOI = 1) A549-ACE2 cells pretreated with IFN-γ with or without IL-1β for 48 h at the highest concentrations (400 U/ml and 10 ng/mL, respectively), and then stained with anti-SARS-CoV-2 nucleocapsid Ab (N; red), DAF-FM (green), and anti-iNOS Ab (cyan), or matching isotype controls (C). Nuclei (blue) were counter-stained with NucBlue. Two or all channels (merge) colocalization profiles are shown. Scale bars, 10 µm. The images are linked to the experiments described in Figure 4C–F.

# Supplementary Tables

| **REAGENT or RESOURCE** | **SOURCE** | **IDENTIFIER** |
| --- | --- | --- |
| Antibodies | | |
| Alexa Fluor 594 anti-NOS2/iNOS | BioLegend | Cat#696804; RRID: AB_2728514 |
| Alexa Fluor 594 rat IgG2bκ isotype | BioLegend | Cat#400661; RRID: N/A |
| Rat monoclonal anti-mouse/human iNOS (clone CXNFT) | eBioscience | Cat#14-5920-82; RRID: AB_2572890 |
| Purified rat IgG2aκ isotype (clone R35-95) | BD Biosciences | Cat#553927; RRID: AB_395142 |
| Goat anti-mouse IgG1 Alexa Fluor 488 | Invitrogen | Cat#A-21121; RRID: AB_2535764 |
| Goat anti-mouse IgG1 Alexa Fluor 568 | Invitrogen | Cat#A-21124; RRID: AB_2535766 |
| Goat anti-rat IgG Alexa Fluor 568 | Invitrogen | Cat#A-11077; RRID: AB_2534121 |
| Goat anti-rat IgG Alexa Fluor 647 | Invitrogen | Cat#A-21247; RRID: AB_141778 |
| Virus strains | | |
| SARS-CoV-2 clinical isolate USA-WA1/2020 | BEI Resources | NR-52281 |
| SARS-CoV-2-mNeonGreen (SARS-CoV-2-mNG) fluorescent clone | World Reference Center of Emerging Viruses and Arboviruses (WRCEVA) at the University of Texas Medical Branch; Xie et al.^25^ | N/A |
| Cell culture | | |
| DMEM, high glucose, HEPES | Gibco | Cat#12430054 |
| DMEM, high glucose, HEPES, no phenol red | Gibco | Cat#21063029 |
| FluoroBrite DMEM | Gibco | Cat#A1896701 |
| Opti-MEM Reduced Serum Medium | Gibco | Cat#31985070 |
| Millicell EZ 4 well glass slide | Millipore | Cat#PEZGS0416 |
| Millicell EZ 8 well glass slide | Millipore | Cat#PEZGS0816 |
| Chemicals and recombinant proteins | | |
| L-NIL (hydrochloride) | Cayman Chemical | Cat#80310; CAS: 159190-45-1 |
| L-NAME (hydrochloride) | Cayman Chemical | Cat# 80210; CAS: 51298-62-5 |
| D-NAME (hydrochloride) | Cayman Chemical | Cat# 21687; CAS: 50912-92-0 |
| Human IFN-α 2a | PBL Assay Science | Cat#11100 |
| Human IFN-β 1b | PBL Assay Science | Cat#11420 |
| Human IFN-λ1 | Peprotech | Cat#300-02L |
| Human IFN-γ | BD Pharmigen | Cat#554617 |
| Human IL-1β | Gibco | Cat#PHC0815 |
| Critical commercial assays | | |
| DAF-FM diacetate | Invitrogen | Cat#D23844 |
| SYTOX Red dead-cell indicator | Invitrogen | Cat#S34859 |
| NucBlue nuclear staining for live cells | Invitrogen | Cat#R37605 |
| ProLong Gold Antifade Mountant | Invitrogen | Cat#P36930 |
| ProLong Gold Antifade Mountant with DNA Stain DAPI | Invitrogen | Cat#P36931 |
| Experimental models: Cell lines | | |
| Simian: Vero E6 cells | ATCC | CRL-1586 |
| Human: Calu-3 cells | ATCC | HTB-55 |
| Human: A549 cell line stably expressing ACE2 (A549-ACE2) | BEI Resources; Laboratory of Bryan Bryson; Klein et al.^23^ | NR-53821 |
| Software and algorithms | | |
| ImageJ (Fiji) | Schindelin et al.^38^ | https://imagej.net/software/fiji/ |
| BioRender: Scientific Image and Illustration Software | BioRender | https://www.biorender.com/ |
| GraphPad Prism software version 9.1.0 | Dotmatics | https://www.graphpad.com/ |
| Leica Microsystems Application Suite | Leica Microsystems | https://www.leica-microsystems.com/products/microscope-software/p/leica-las-x-ls/ |
| FlowJo software version 10.7.1 | BD Biosciences | https://www.flowjo.com/solutions/flowjo/downloads |
| FACSDiva software version 8.0.2 | BD Biosciences | https://www.bdbiosciences.com/en-us/products/software/instrument-software/bd-facsdiva-software |
| Spearman-Karber method | Ramakrishnan MA^34^ | https://www.wjgnet.com/2220-3249/full/v5/i2/85.htm |
| Special Order Research Equipment | | |
| Leica DMi1 inverted phase contrast digital microscope | Leica Microsystems; UCLA BSL3 high-containment facility | https://www.leica-microsystems.com/products/light-microscopes/p/leica-dmi1/ |
| Leica DM IRB inverted modulation contrast microscope | Leica Microsystems; UCLA BSL3 high-containment facility | https://www.leica-microsystems.com/products/light-microscopes/p/leica-dm-il/ |
| Leica TCS SP8 Digital LightSheet Laser Scanning Confocal Microscope | Leica Microsystems; ALMS/CNSI | https://www.leica-microsystems.com/products/confocal-microscopes/p/leica-tcs-sp8-dls/ |
| LSRII Analytic Flow Cytometer | BD Biosciences; UCLA Flow Cytometry Core Facility | https://www.bd.com/resource.aspx?IDX=17868 |
| LSRFortessa X-20 | BD Biosciences; UCLA Flow Cytometry Core Facility | https://www.bdbiosciences.com/en-us/products/instruments/flow-cytometers/research-cell-analyzers/bd-lsrfortessa-x-20 |

**Supplementary Table 1.** Key reagents and resources used in this study.

**Supplementary Table 2.** Hillslopes and half maximal inhibitory concentration (IC_50_) of SARS-CoV-2 replication in Vero E6 cells treated with IFN-γ. Vero E6 cells were pretreated with increasing concentrations of IFN-γ (50, 100, 200, 400 U/mL), IL-1β (1.25, 2.5, 5, or 10 ng/mL), or IFN-γ in combination with IL-1β (50/1.25, 100/2.5, 200/5, or 400/10, respectively) for 48 h, infected with SARS-CoV-2-mNG (MOI = 0.1), and analyzed flow cytometry. FlowJo software was used to determine the % of Vero E6 cells infected with SARS-CoV-2-mNG. Data are means ± SEM (*n* = 5). Dose-response curves of SARS-CoV-2-mNG signal inhibited by cytokines treatment were then generated, and a nonlinear regression model (inhibitor vs. normalized response – variable slope) on GraphPad Prism software was applied to calculate the IC_50_ values and Hillslopes. The data are linked to the experiments described in Figure 1C–E.
